# Supplementary figures and images for: Whole-chromosome hitchhiking driven by a male-killing endosymbiont
Source: PLoS Biol. 2020 Feb 27;18(2):e3000610. doi: 10.1371/journal.pbio.3000610 (PMC7046192; doi:10.1371/journal.pbio.3000610)

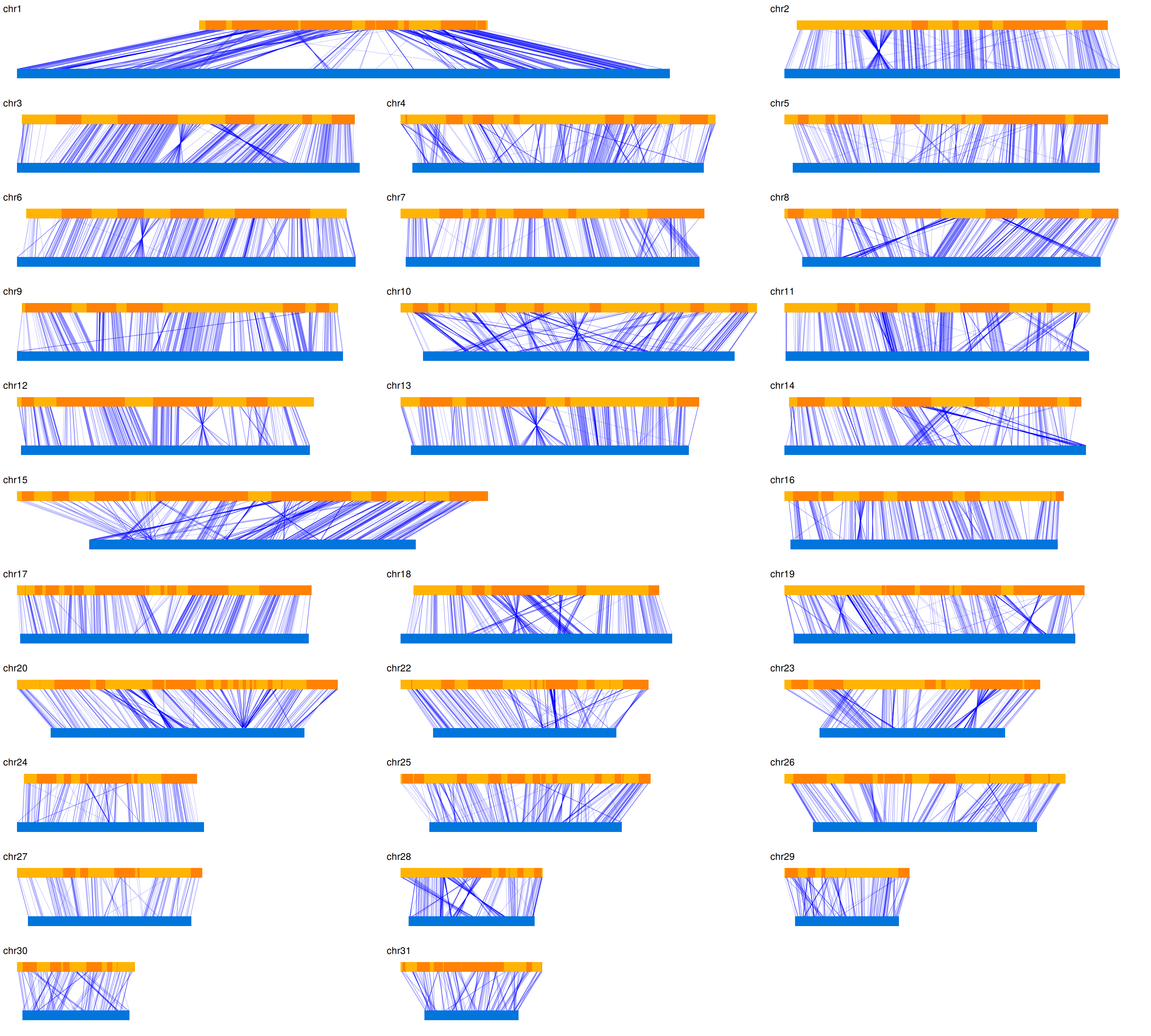

Supplement: S1 Fig — Homology with the H. melpomene genome (corrected for known fusion events [9,30,32] and scaffolded into chromosomes [31]) (blue) allowed us to construct a robust pseudo-chromosomal assembly for D. chrysippus. Scaffolds of D. chrysippus are shown in alternating shades of orange. Blue lines connect homologous genes (BLAST E-value < 1 × 10^−20^, identity >50%). Data deposited in the Dryad repository [36]. (PNG) [file pbio.3000610.s001.png]

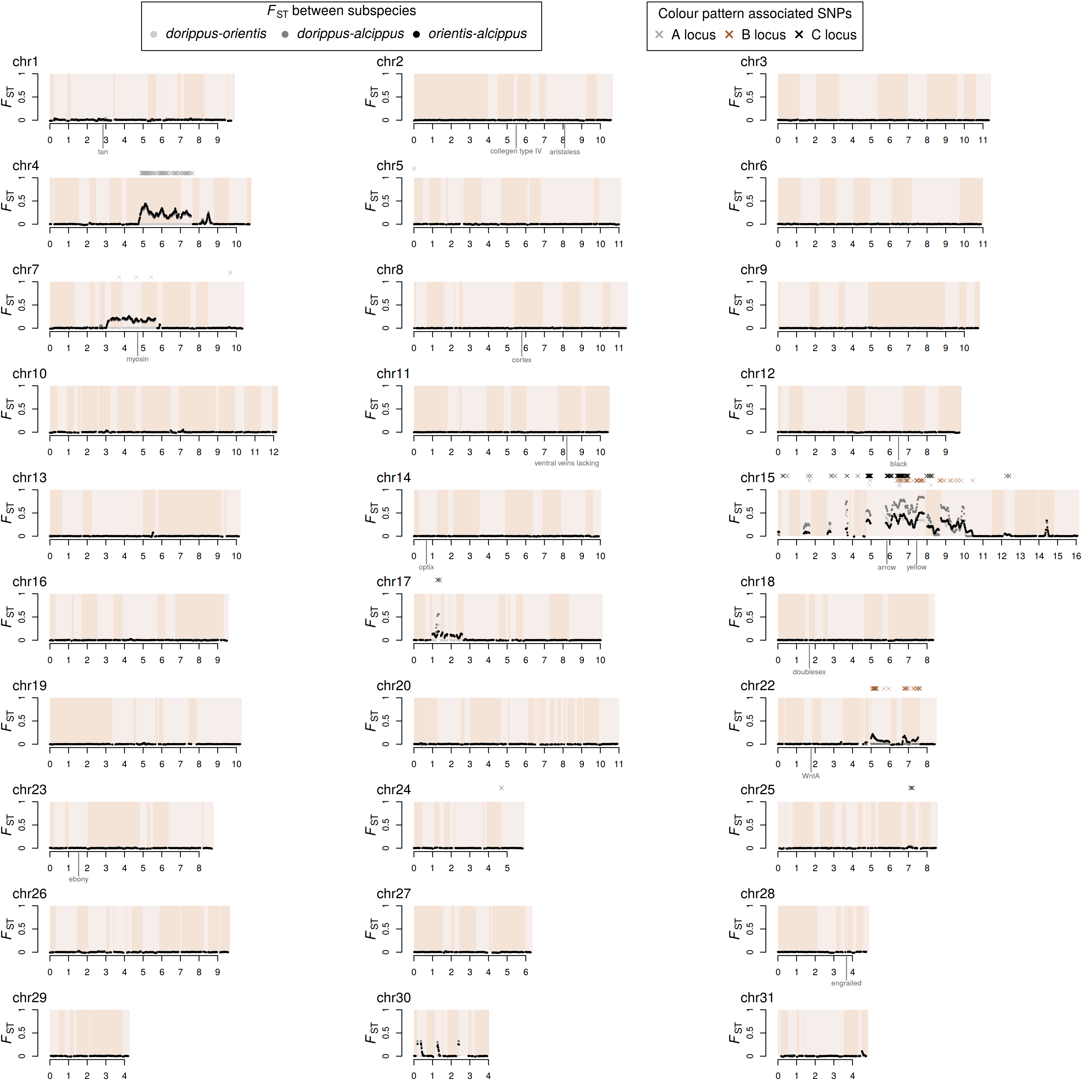

Supplement: S2 Fig — FST is plotted across each chromosome between three different subspecies of D. chrysippus, as indicated above the plot. Scaffolds are indicated by light and dark shading. Numbers on the x-axis indicate chromosome position in Mb. Coloured crosses above the plots indicate SNPs strongly associated with the phenotypes controlled by the A (grey), B (brown), and C (black) loci (Wald test, 99.99% quantile). A number of candidate genes are annotated on the plot. These include known and putative wing patterning genes in Heliconius (optix [84], cortex [85], WntA [40], aristaless [86], and ventral veins lacking [42]) and Papilio spp. (doublesex [87] and engrailed [88]). A myosin gene thought to be associated with a pale mutant form in D. plexippus [69] is also indicated, along with collegen type IV, which was found to be associated with migratory behaviour in D. plexippus [69]. Several melanism-related genes are also annotated, as well as arrow, which was added to the list of candidates post hoc due to strong association with colour pattern (see main text). Of our a priori candidates, only yellow is found to associate with colour pattern in D. chrysippus. Data deposited in the Dryad repository [36]. (PNG) [file pbio.3000610.s002.png]

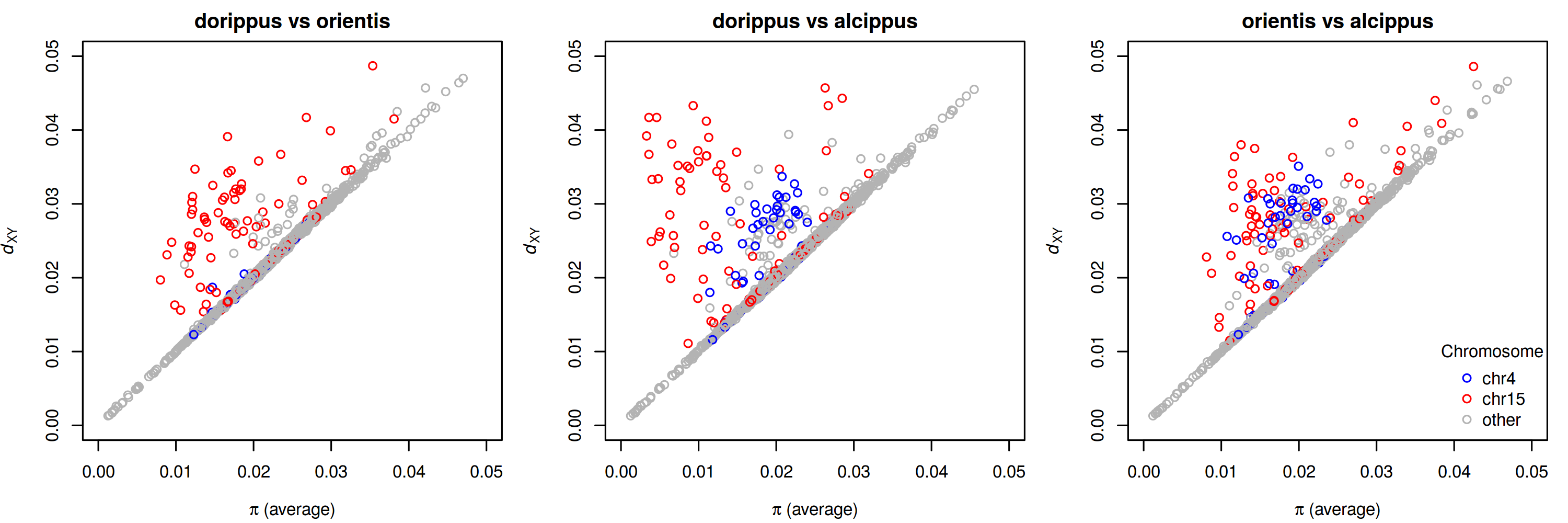

Supplement: S3 Fig — Absolute divergence between each pair of populations (dXY) and nucleotide diversity within populations (π) were computed for nonoverlapping 100-kb windows. The value of π plotted is the average between the two populations in each plot. The clustering of points along the diagonal indicates that diversity within each subspecies is similar to divergence between subspecies, consistent with a single nearly panmictic population. Points that deviate to the left of the diagonal indicate either excess divergence between subspecies or reduced diversity within subspecies, or both. Here, the colour pattern–associated regions on Chromosomes 4 and 15 (indicated in colour for convenience) show signatures of local adaptation with both reduced within-population diversity and increased between-population divergence, as would be expected if selection limits effective gene flow at these loci. One pair of populations, D. c. dorippus and D. c. orientis, are diverged at chr15 but not Chromosome 4, which is also expected as they only differ in their forewing phenotype and both lack the white hindwing patch. Data deposited in the Dryad repository [36]. chr15, Chromosome 15. (PNG) [file pbio.3000610.s003.png]

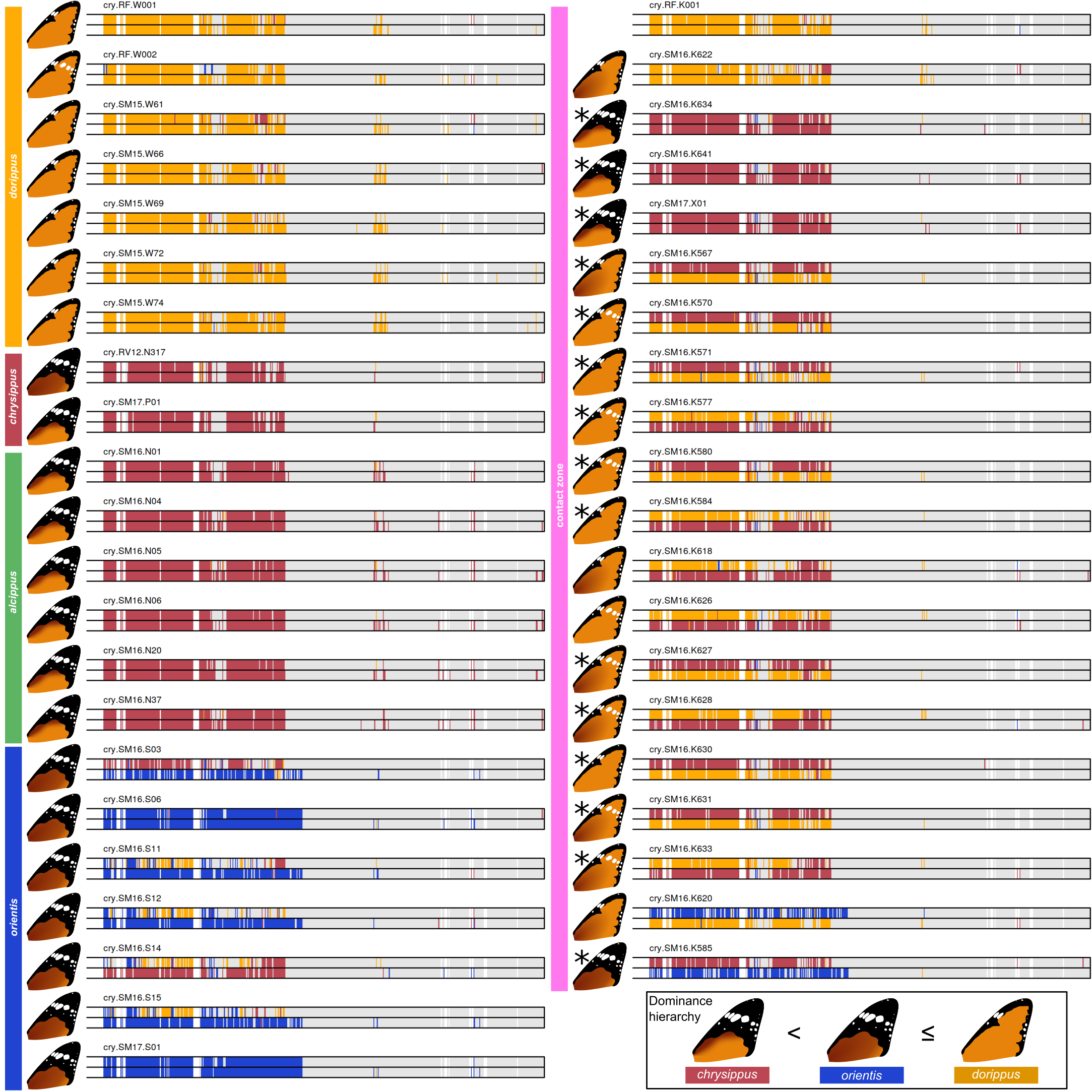

Supplement: S4 Fig — Coloured blocks indicate 20-kb windows in which sequence haplotypes could be clustered into one of three genetic clusters (yellow: dorippus, red: chrysippus/alcippus, blue: orientis) based on pairwise genetic distances (see Methods for details). Windows in grey show insufficient relative divergence to be assigned to a cluster. White gaps indicate missing data. There are three clearly distinct alleles that correspond largely with colour pattern. Heterozygotes indicate a dominance hierarchy: The BCdorippus allele (yellow) is the most dominant and produces the dorippus phenotype (no black forewing tip). Around half of the heterozygotes with one copy of the dorippus allele express the transiens phenotype, with white marks on the forewing. The BCorientis allele (blue) corresponds with the orientis phenotype (black wing tip and dark background colour). It is dominant over the BCchrysippus allele, which produces the chysippus phenotype (black wing tip with light background colour) only when homozygous. There is evidence of recombination in the form of mosaic haplotypes. Finally, samples found to be carrying the neo-W chromosome (see main text) are indicated with an asterisk. All carry the BCchrysippus allele. Note that no phenotype was recorded for the reference genome individual RF.K001. Data deposited in the Dryad repository [36]. chr15, Chromosome 15. (PNG) [file pbio.3000610.s004.png]

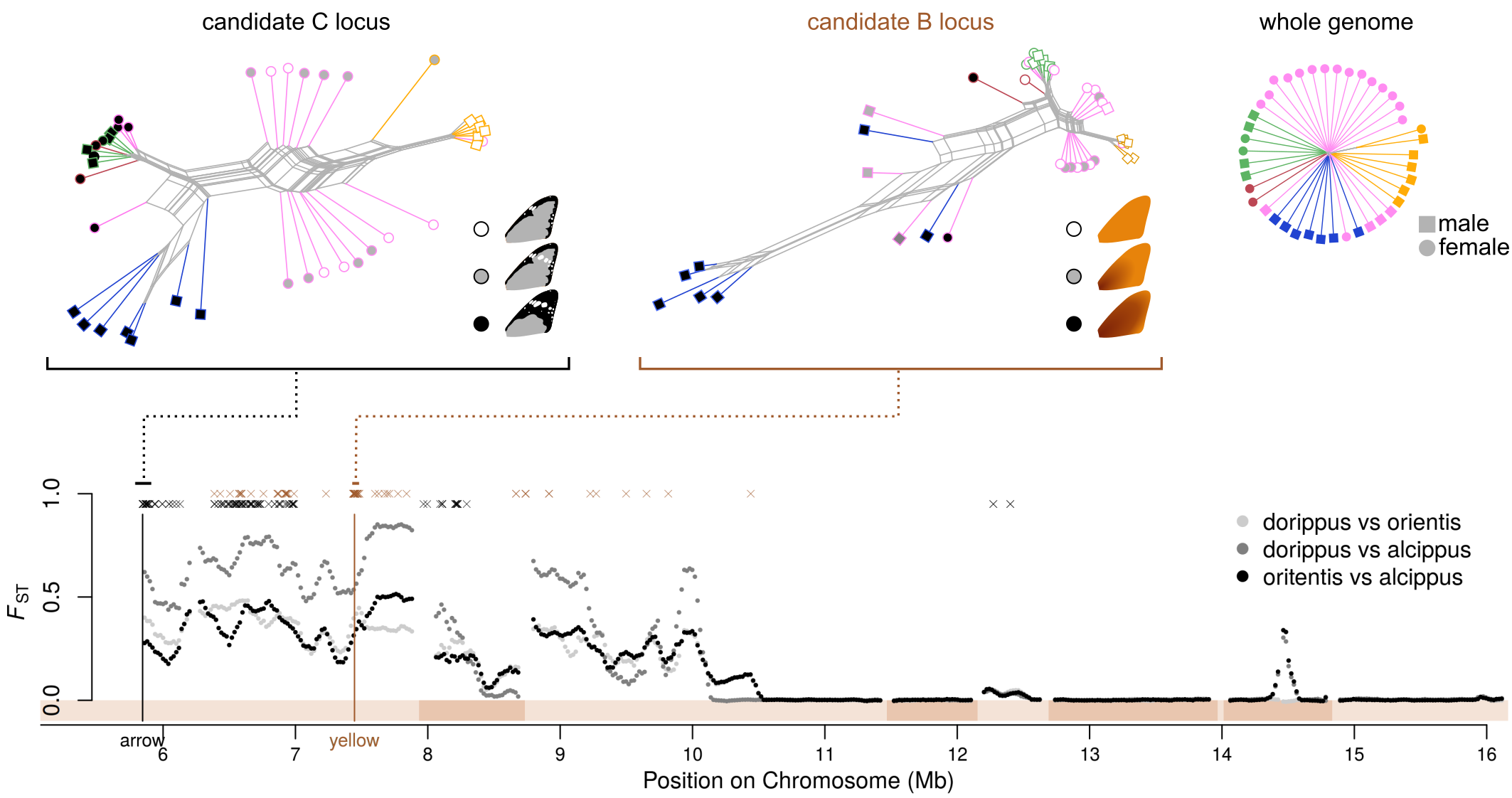

Supplement: S5 Fig — Differentiation (FST) is plotted across part of chr15 (bottom). Above the plot, locations of SNPs most strongly associated with the B and C loci (Wald test, 99.99% quantile) are shown: ‘B locus’ (controlling brown/orange background) in brown and ‘C locus’ (controlling forewing black tip) in black. The best respective candidate genes, yellow and arrow, are indicated on the plot. At the top, distance-based phylogenetic networks constructed for regions around the candidate genes (30 kb around yellow and 100 kb around arrow) are shown. Colours indicate subspecies as in Fig 1A, and shapes indicate sex. Phenotypes are coded black and white for putative homozygotes and grey for putative heterozygotes. A corresponding network for the whole genome is included for comparison, showing how undifferentiated the subspecies are in general. Data deposited in the Dryad repository [36]. chr15, Chromosome 15. (PNG) [file pbio.3000610.s005.png]

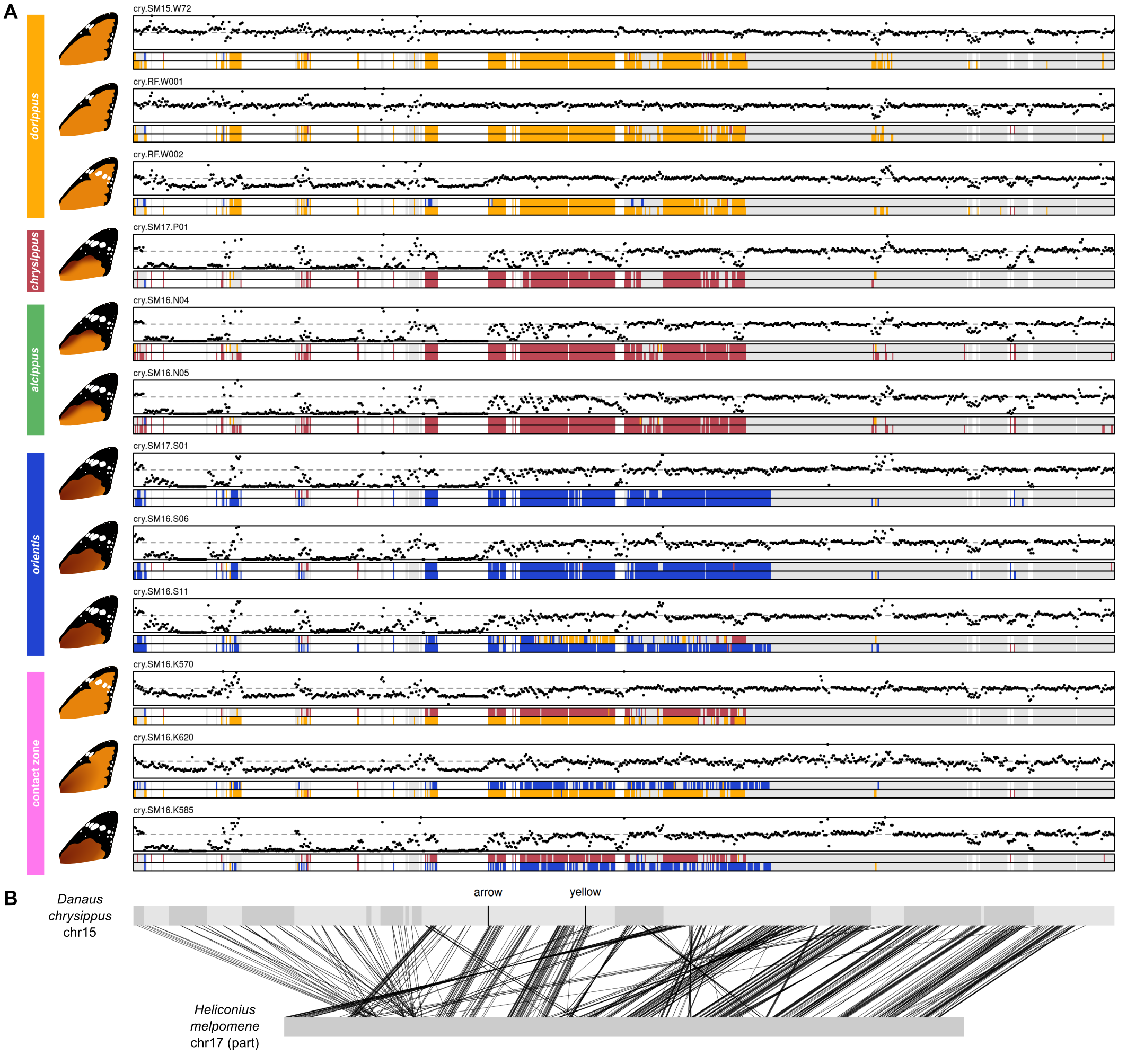

Supplement: S6 Fig — (A) Dots indicate median read coverage in 20-kb windows across chr15, normalised relative to the genome-wide mean (dashed line). Twelve representative individuals are shown. All individuals fall into one of three categories: normal coverage, approximately half coverage, or approximately zero coverage across the first third (5.84 Mb) of the chromosome, indicating an insertion polymorphism that is either homozygous present/absent or heterozygous. Coloured blocks indicate allelic clustering for each 20-kb window (see S4 Fig), with white indicating gaps in the alignment because of variable sequence coverage. (B) Comparison of homologous genes in the H. melpomene genome indicates several genes near the proximal end of the chromosome that are duplicated multiple times in our D. chrysippus reference genome. Locations of the candidate B and C genes yellow and arrow (see S5 Fig) are indicated. Scaffolds in the D. chrysippus pseudo-chromosomal assembly are alternately shaded light and dark. Data deposited in the Dryad repository [36]. chr15, Chromosome 15. (PNG) [file pbio.3000610.s006.png]

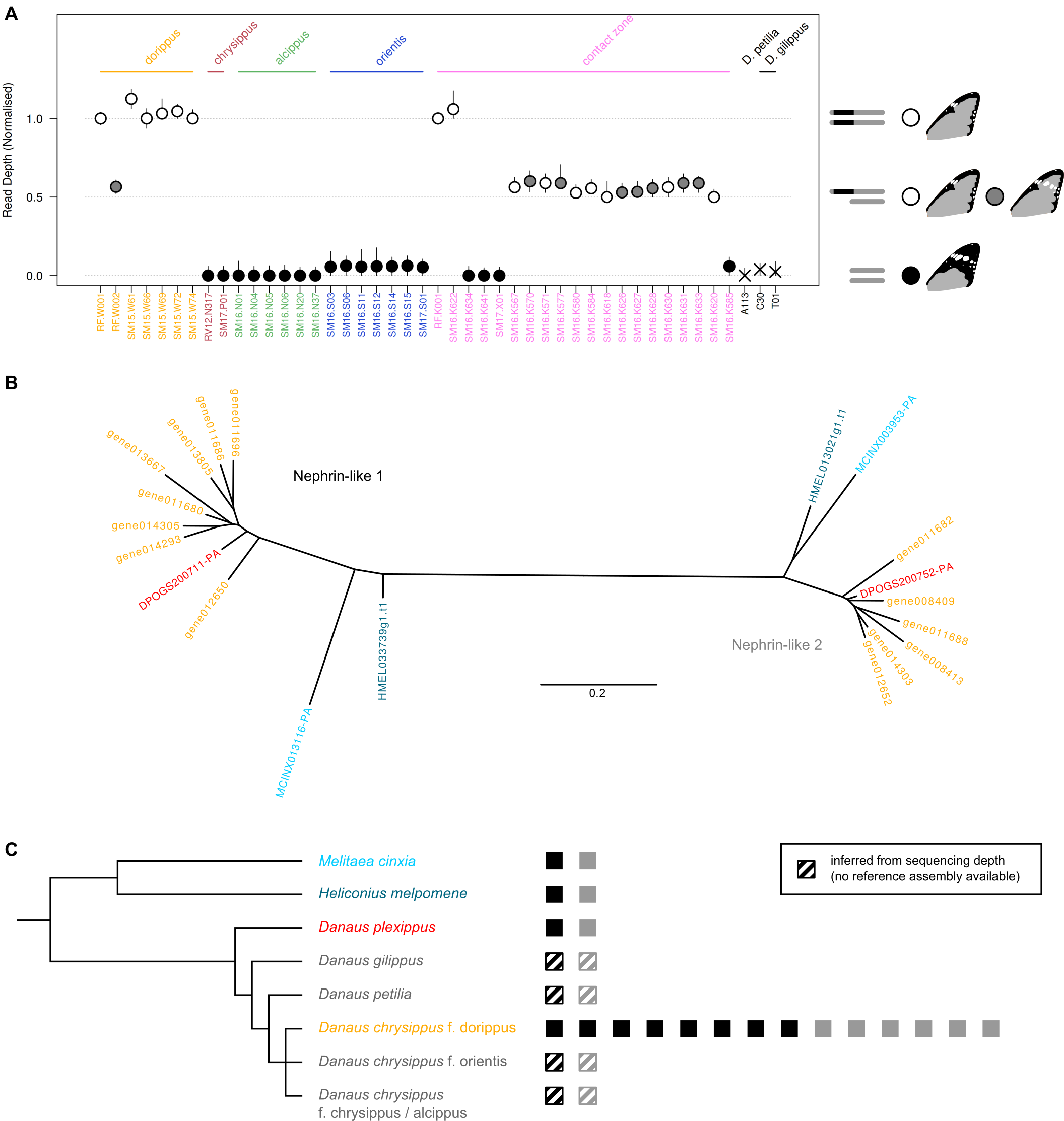

Supplement: S7 Fig — (A) Depth of coverage across the expansion region (see S6 Fig), in each individual, normalised by the genome average. Points represent the median coverage over 20-kb windows, and vertical lines indicate the 25% and 75% quantiles. Homozygous individuals with two copies of the expansion have a normal depth of approximately 1, heterozygous individuals have a depth of approximately 0.5, and those homozygous for a lack of the expansion have a depth of approximately 0. There is perfect correspondence between presence of the expansion and the dorippus phenotype (lack of black forewing tip). Heterozygotes display either the dorippus pattern or the transiens pattern, with white marks on the forewing, consistent with the approximately 50% penetrance described in previous crosses [89]. (B) Maximum likelihood phylogeny of Nephrin-like protein sequences encoded by two genes located within the expansion region. Homologous genes from Danaus plexippus, H. melpomene, and Melitaea cinxia are included. The tree indicates that the ancestral state in the Nymphalidae is to have two copies of the gene, while the D. chrysippus assembly has 14 copies (8 and 6, respectively). (C) The number of copies of nephrin-like 1 and 2 is indicated in black and grey, respectively. Although we have just one assembly from a sample homozygous for the BCdorippus allele, the read-depth data (see panel A and S6 Fig) suggest that the other D. chrysippus morphs have the ancestral state, lacking the additional copies, as do the two outgroup species: D. petilia and D. gilippus. Data deposited in the Dryad repository [36]. chr15, Chromosome 15. (PNG) [file pbio.3000610.s007.png]

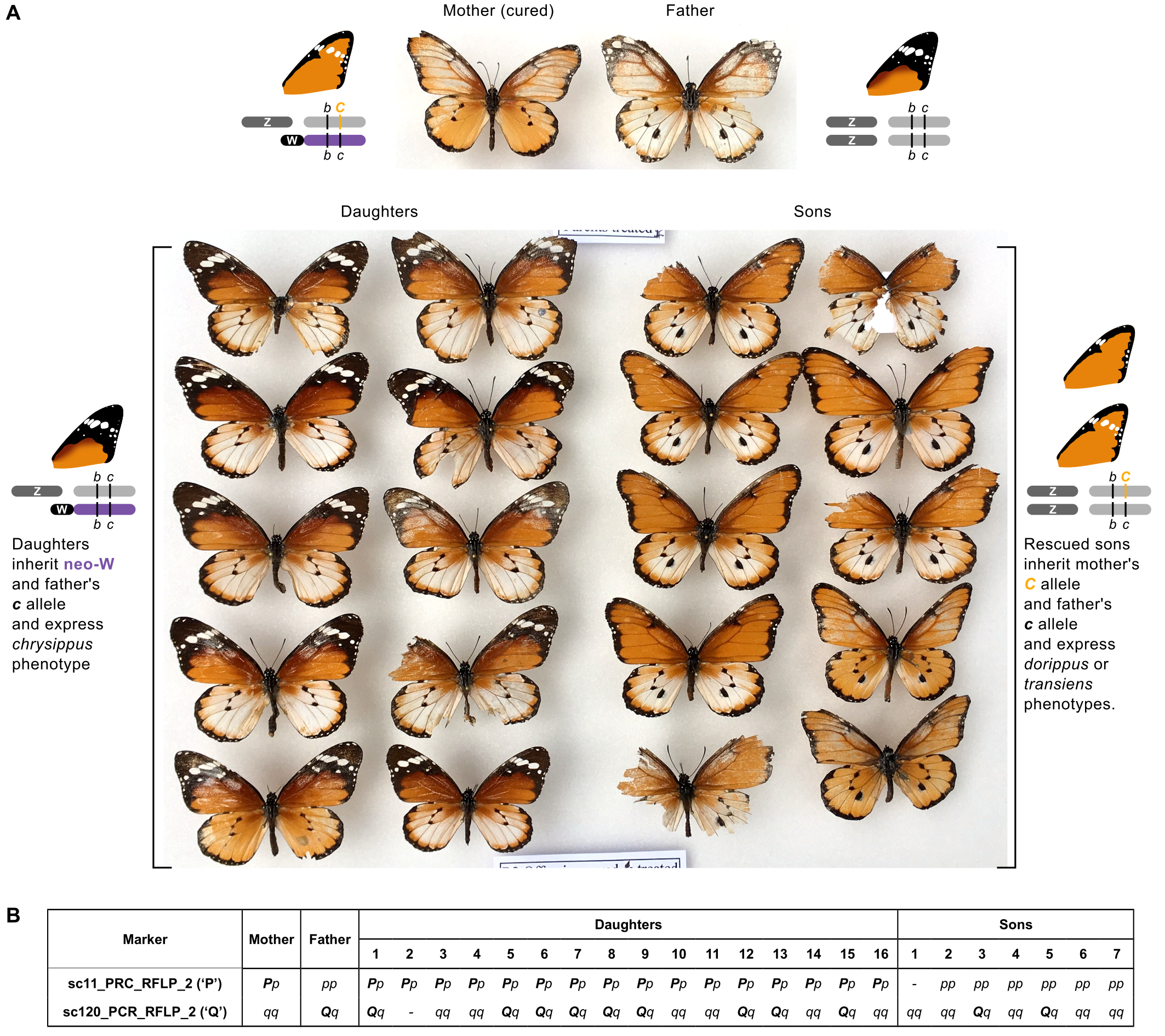

Supplement: S8 Fig — (A) Sex linkage of forewing pattern controlled by the BC supergene. A female descending from the contact zone (top left) was cured of Spiroplasma. Her transiens phenotype indicated that she was heterozygous Cc (Fig 1B). She was crossed with a cc male (black forewing tips) to produce the F1 brood shown. Male offspring (right) who would ordinarily have been killed by Spiroplasma expressed the dorippus (or transiens) phenotype without black forewing tips, indicating that they had all inherited the C allele from their mother (note that males can be identified by the additional large black spot on the hindwing). Female offspring (left) all expressed the chrysippus phenotype, indicating that they had inherited the recessive c allele from both parents. (B) Inheritance of two chr15 PCR markers (here designated P and Q) was tracked in the F5 brood of the cured line. One marker (‘P’) was heterozygous in the mother and showed complete sex linkage. The other marker (‘Q’) was heterozygous in the father and segregated independently of sex. These results are consistent with chr15 forming a neo-W in the mother, while both copies of the father’s chr15 are autosomal. chr15, Chromosome 15. (PNG) [file pbio.3000610.s008.png]

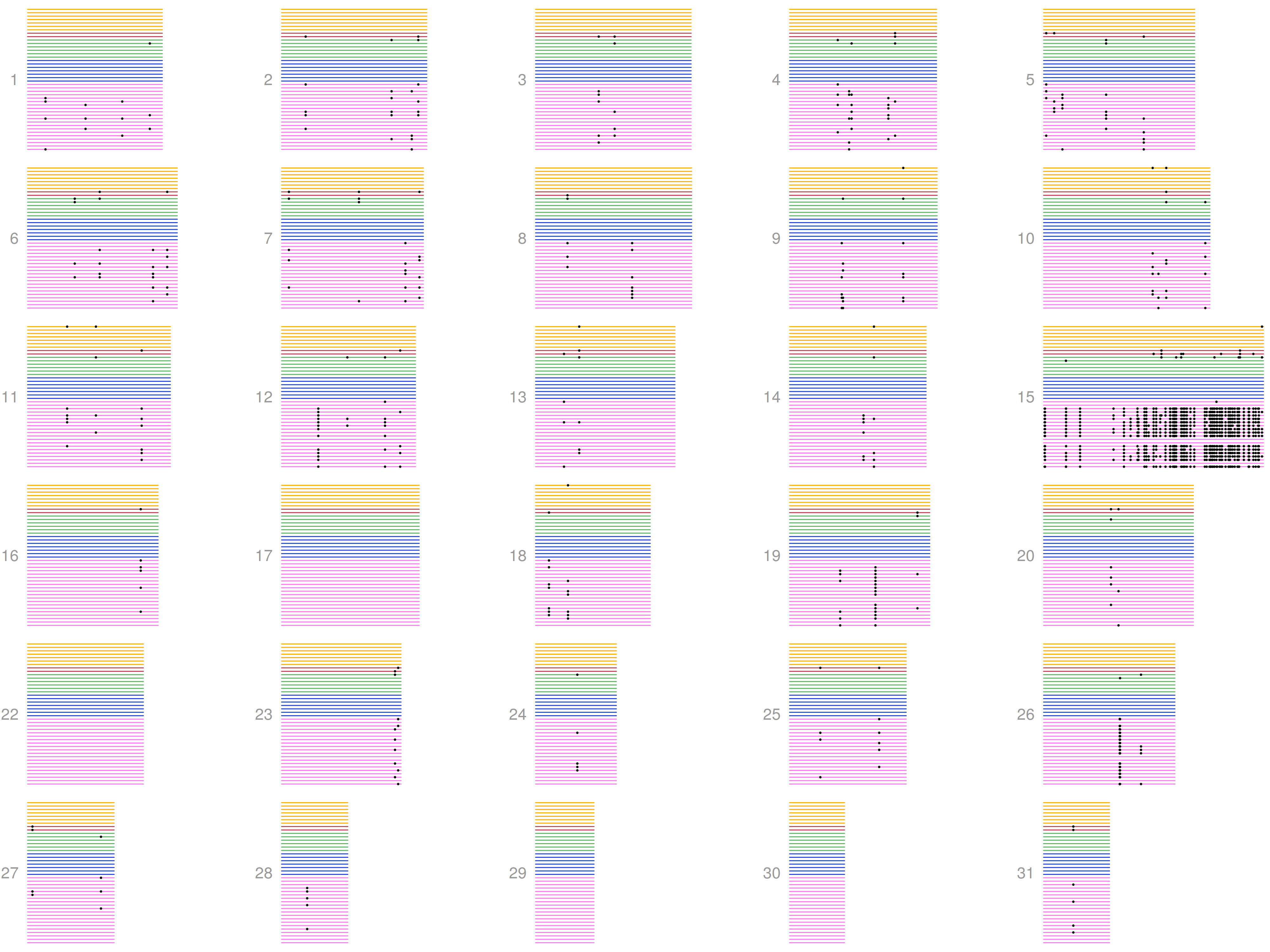

Supplement: S9 Fig — The 30 chromosomes are shown with each line representing an individual, coloured according to population: yellow = D. c. dorippus, red = D. c. chrysippus, green = D. c. alcippus, blue = D. c. orientis, pink = contact zone. Black points indicate the location of mutations shared by at least four females and absent from males. These are strongly clustered on chr15 and shared by a group of contact zone females, indicating that a conserved neo-W haplotype is shared by this female lineage. The noticeable absence of mutations on the proximal (left) region of chr15 reflects the large sequencing gaps corresponding to the expansion cluster in the BCdorippus allele (see S6 Fig). Data deposited in the Dryad repository [36]. (PNG) [file pbio.3000610.s009.png]

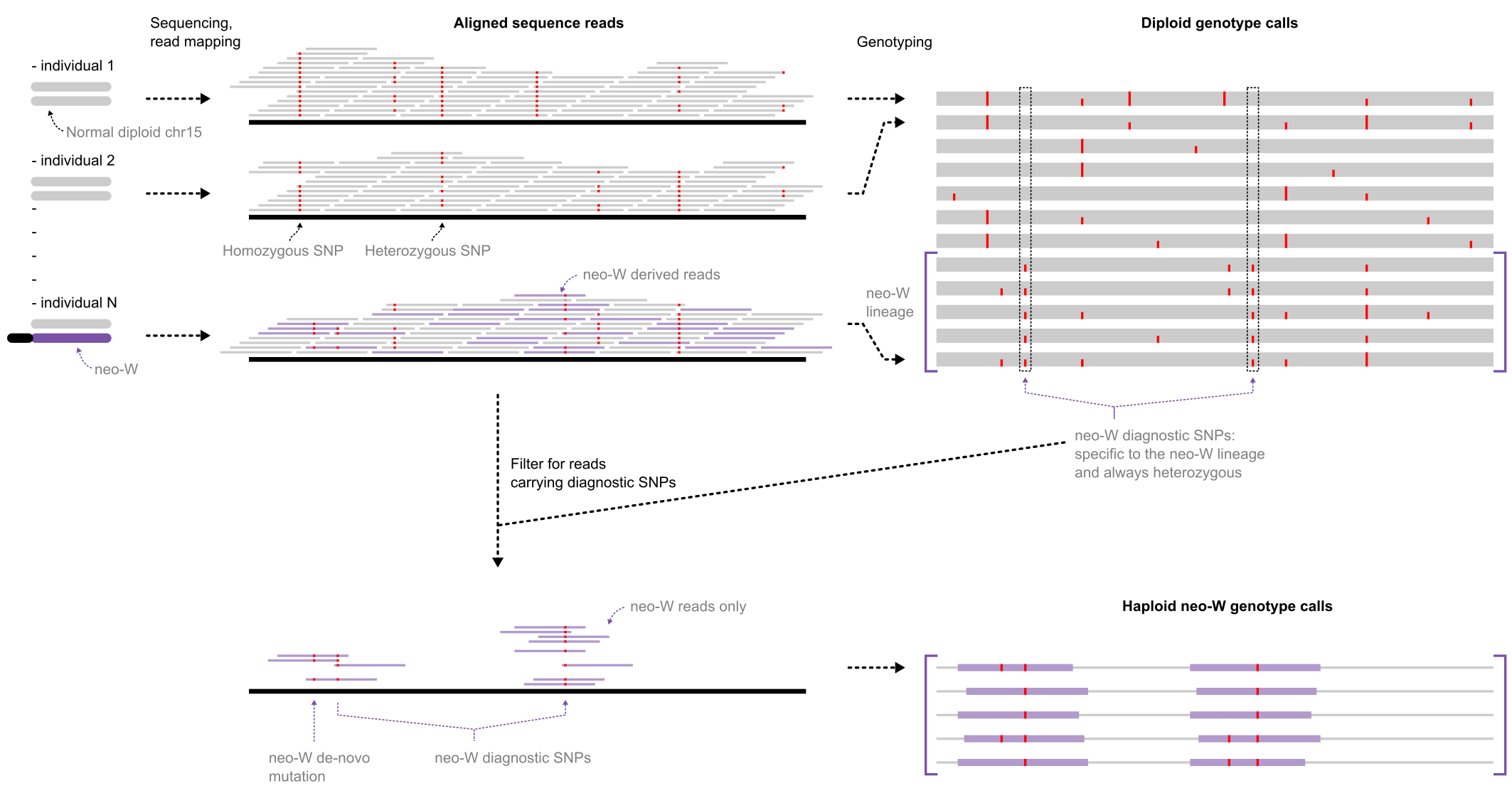

Supplement: S10 Fig — Schematic representation of the bioinformatic pipeline to isolate the neo-W haplotype from unphased resequencing data. Due to the recency of its formation, sequencing reads from the neo-W are not significantly divergent and will therefore map to the reference genome chr15. The challenge is to separate reads that derive from the neo-W and autosomal haplotypes, despite them all mapping to the same parts of the reference genome. Our solution is to use diagnostic mutations that are unique to the neo-W haplotype and shared by the multiple individuals that carry the neo-W. We identified candidate mutations specific to the neo-W haplotype as those at which all 15 females in the neo-W lineage are heterozygous, while all 27 remaining individuals are homozygous. We then used these candidate neo-W specific mutations to extract sequence reads that are specific to the neo-W. These represent only a fraction of the chromosome, because they represent only the reads carrying diagnostic mutations and their paired-end partners. The identification of these neo-W specific reads allows the identification of additional mutations on the same read that occurred after the formation of the neo-W. These can be used to estimate genetic diversity across the neo-W (accounting for the large amount of missing data) and also to infer a genealogy for the neo-W. (PNG) [file pbio.3000610.s010.png]

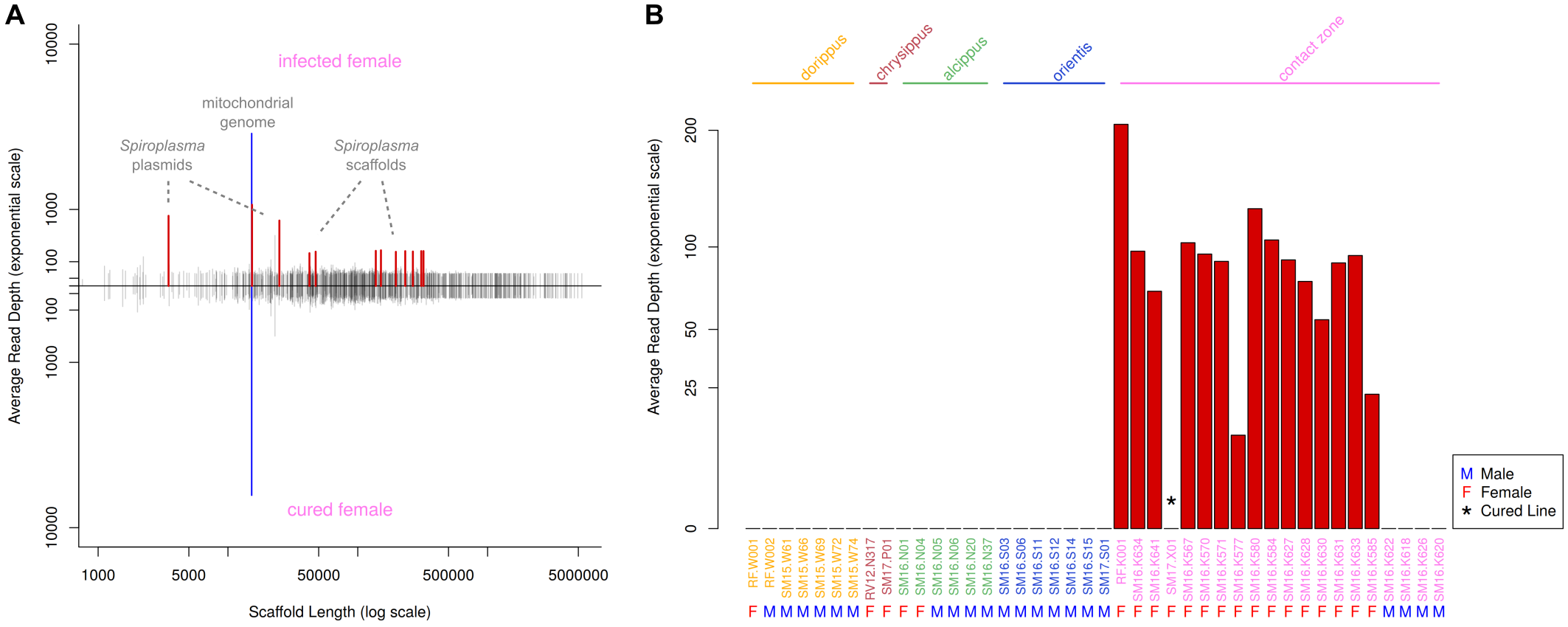

Supplement: S11 Fig — (A) Sequencing read depth of coverage averaged by scaffold (y-axis, exponential scale) and plotted against scaffold length (x-axis, log scale). Depth is shown for a suspected infected female above and a female from the tetracycline-treated ‘cured line’ below. Scaffolds identified as belonging to the Spiroplasma genome are shown in red. The mitochondrial genome is shown in blue. (B) Bars show the average depth of reads mapping to the Spiroplasma genome for each resequenced D. chrysippus individual. Note that all females from the hybrid zone are found to be infected, with the exception of the single individual from the cured line. Data deposited in the Dryad repository [36]. (PNG) [file pbio.3000610.s011.png]

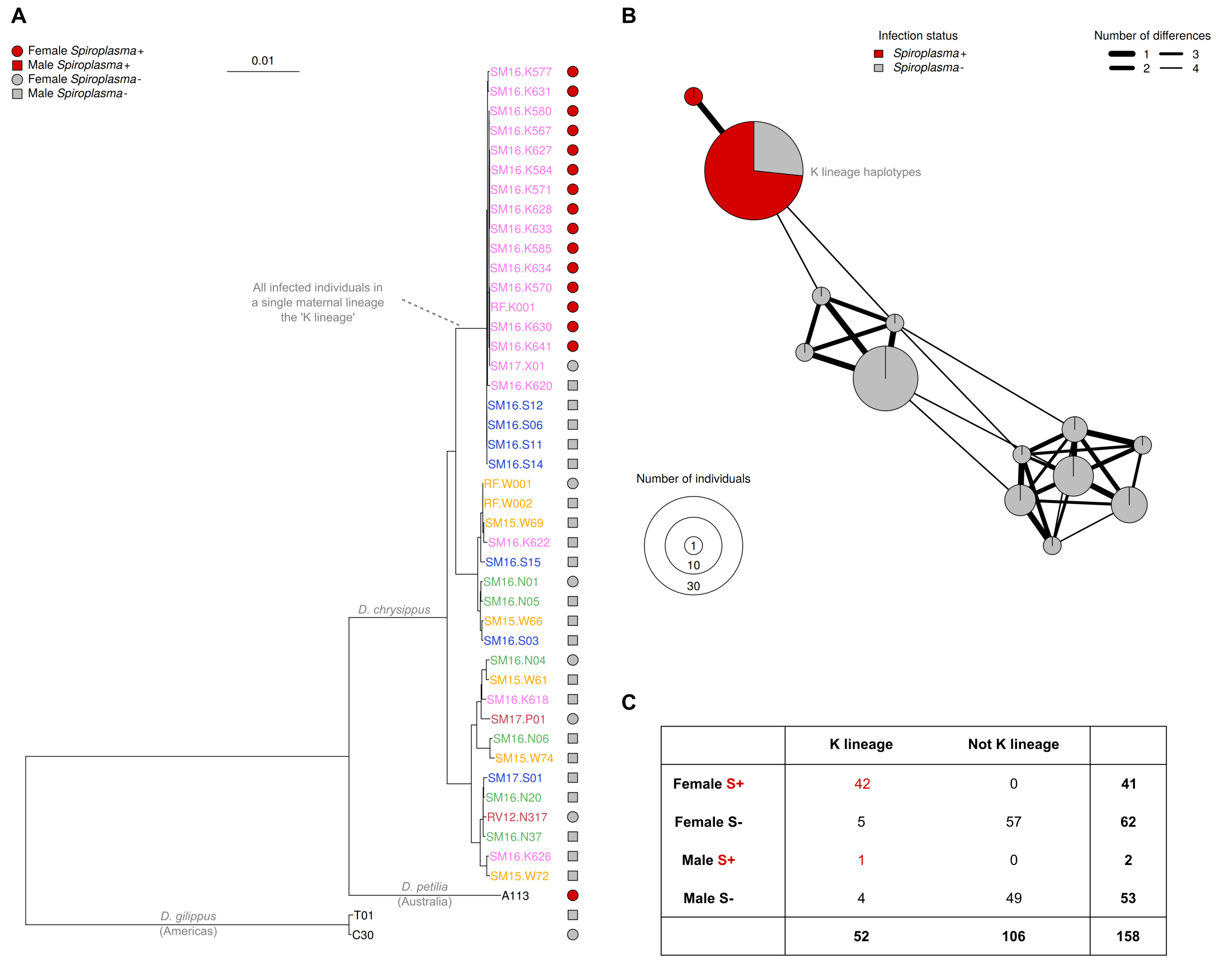

Supplement: S12 Fig — (A) A whole mitochondrial maximum-likelihood phylogeny for the 42 resequenced individuals indicates that all infected D. chrysippus females belong to a single mitochondrial clade (here called the K lineage), consistent with strict matrilineal inheritance of Spiroplasma. Note that the single D. petilia male from Australia was found to be infected by a related Spiroplasma strain but has a different mitochondrial haplotype, indicating an independent infection. (B) COI haplotype network for 66 individuals further supports the finding that only K lineage individuals are infected. (C) A PCR assay (see S11 Table) for an SNP specific to the K lineage applied to 158 individuals further confirms the finding that only the K lineage carries the infection. Note that one male was found to be infected, probably representing a rare survivor from an infected mother, as has been observed in some experimental crosses [23]. Data deposited in the Dryad repository [36]. COI, Cytochrome Oxidase Subunit I. (PNG) [file pbio.3000610.s012.png]

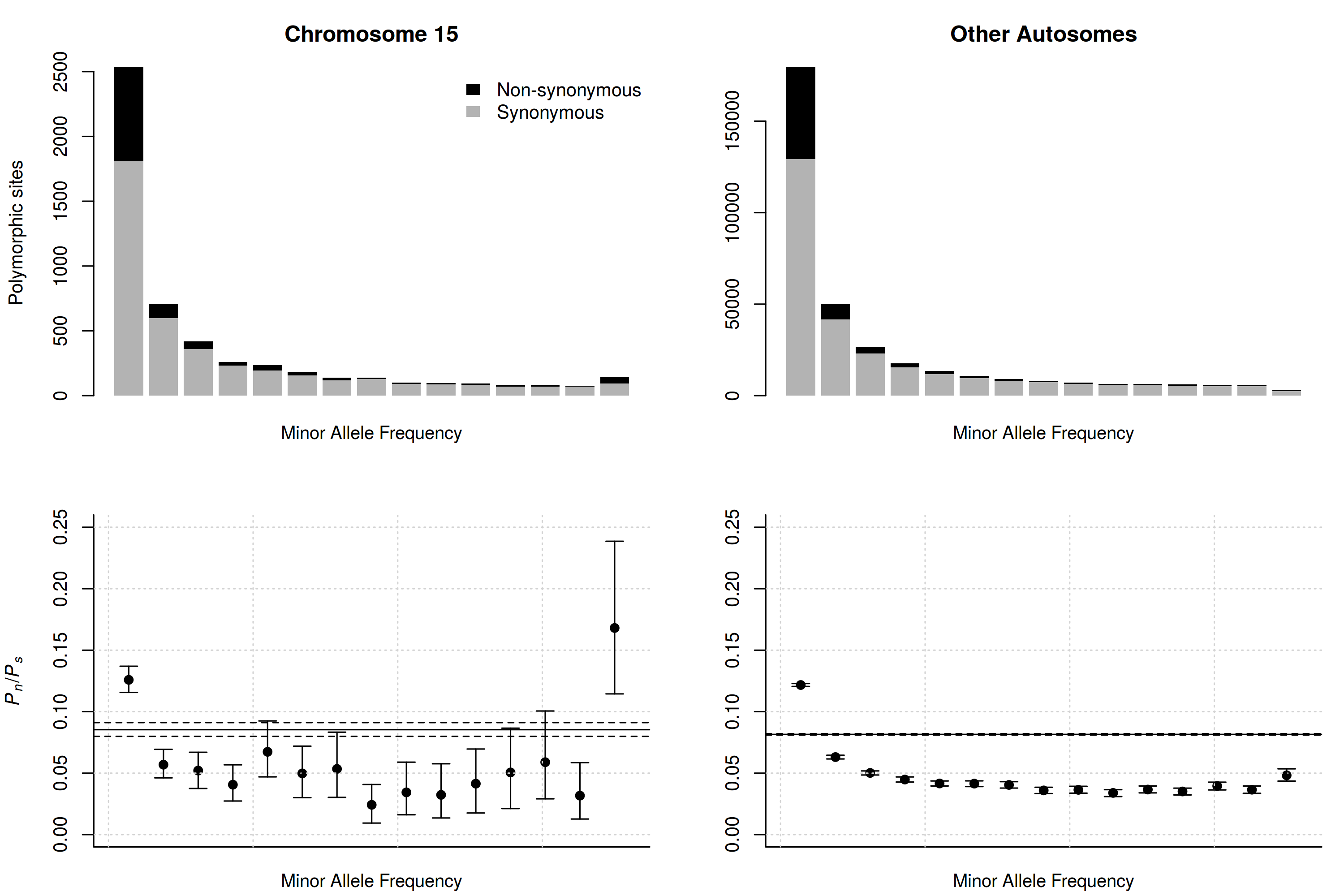

Supplement: S13 Fig — Barplots(top) show the frequency distribution of synonymous (grey) and non-synonymous (black) polymorphisms in the neo-W lineage (i.e., contact-zone females carrying the neo-W chromosome). Values for chr15 are shown on the left and combined values across all other autosomes are shown on the right. Below, Pn/Ps (the normalised ratio of non-synonymous to synonymous polymorphisms) is shown for each frequency class. Error bars show the 95% confidence interval based on 1,000 bootstrap replicates. These plots show that non-synonymous polymorphisms are generally skewed toward lower frequency but that chr15 carries a significant excess of non-synonymous polymorphisms at high frequency in the population. This is consistent with hitchhiking of previously rare mildly deleterious alleles to high frequency on the neo-W. Data deposited in the Dryad repository [36]. chr15, Chromosome 15. (PNG) [file pbio.3000610.s013.png]

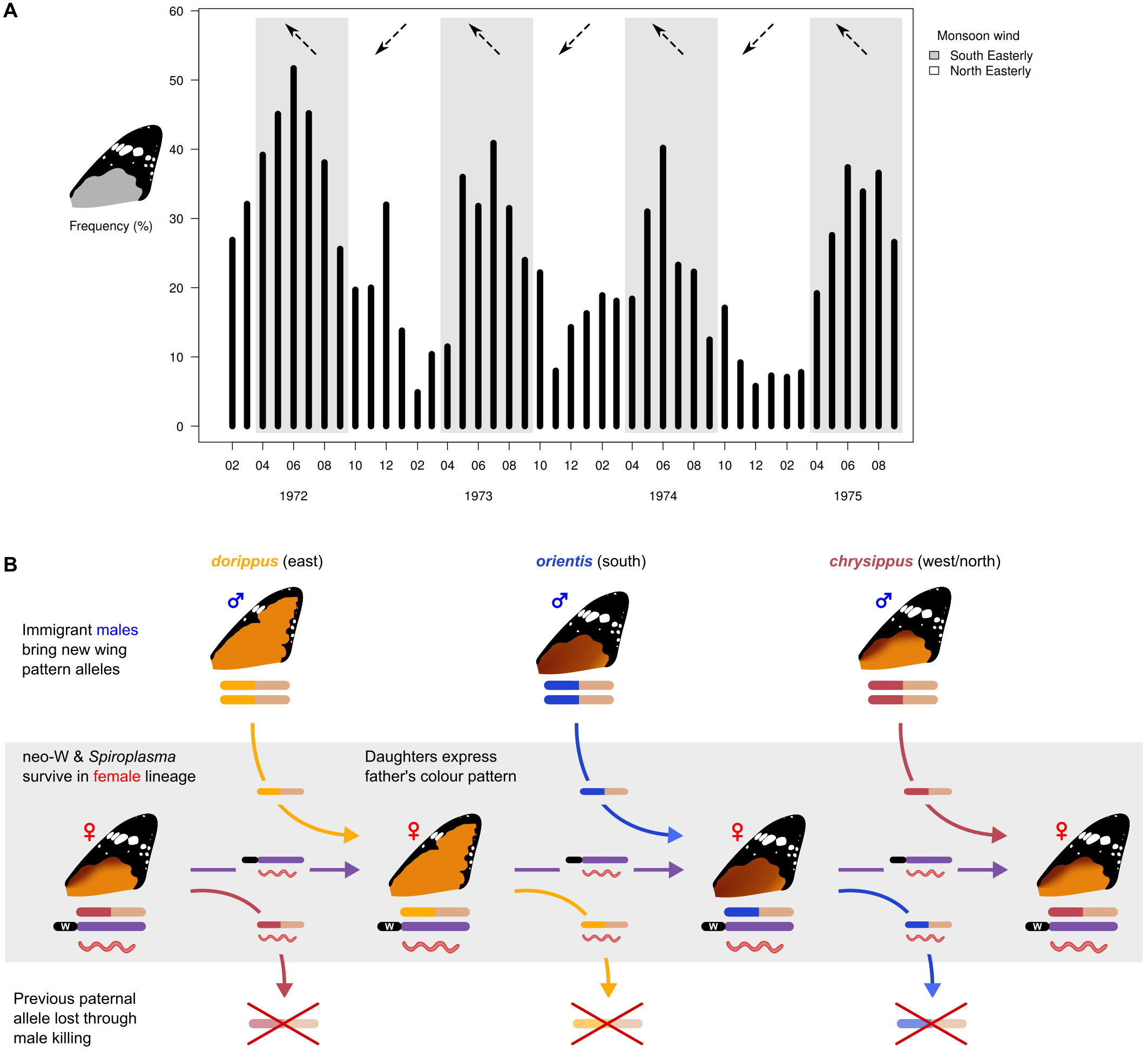

Supplement: S14 Fig — (A) Average monthly frequencies of the black forewing phenotype (cc genotype, BCorientis and BCchrysippus alleles) show how immigration of different subspecies into the contact zone varies seasonally (data from Smith and colleagues [16], collected at Dar es Salaam between 1972 and 1975). (B) Phenotypes of females carrying the neo-W and Spiroplasma depend on the source of immigrant males (top row). Each generation, females (middle row) inherit both the neo-W and Spirplasma from their mother, and an autosomal chr15 copy from their immigrant father. The neo-W is recessive, causing these females to express their father’s phenotype. After persisting in the female for one generation, the autosomal chr15 copy carrying the paternal allele is lost through male-killing, i.e., a genetic sink (bottom row). The progression from left to right illustrates how seasonal changes in the predominant source of immigrant males can drive corresponding changes in the phenotypes of the contact zone females. Data deposited in the Dryad repository [36]. chr15, Chromosome 15. (PNG) [file pbio.3000610.s014.png]
