# Supplementary material for: Whole-chromosome hitchhiking driven by a male-killing endosymbiont
Source: PLoS Biol. 2020 Feb 27;18(2):e3000610. doi: 10.1371/journal.pbio.3000610 (PMC7046192; doi:10.1371/journal.pbio.3000610)
Supplement: S1 Table — (PDF) [file pbio.3000610.s015.pdf]

S1 Table. Sequence data used for reference genome assembly

Library	Type	Read pairs passed (million)	Read pairs trimmed (million)	Mate-pairs	Paired-end	Unknown	Single end	Insert size exp. (Kb)	Insert size obs. (Kb)	Total Gb	Est. depth of cov.
LIB23244	PE	170.64	164.84		100%			0.5		82.42	329.6
LIB23234	MP	18.43	18.43	70.63%	17.32%	6.53%	5.52%	11	11.5	9.21	36.85
LIB23235	MP	30.88	30.87	74.71%	16.35%	3.88%	5.07%	9.5	9.5	15.44	61.74
LIB23236	MP	30.36	30.35	75.50%	15.91%	3.48%	5.11%	8	8.5	15.18	60.7
LIB23239	MP	49.53	49.51	76.88%	14.17%	3.60%	5.34%	5	4.2	24.76	99.02
LIB23242	MP	42.81	42.80	76.31%	15.29%	4.07%	4.32%	2	2	21.40	85.6
