# Supplementary material for: Whole-chromosome hitchhiking driven by a male-killing endosymbiont
Source: PLoS Biol. 2020 Feb 27;18(2):e3000610. doi: 10.1371/journal.pbio.3000610 (PMC7046192; doi:10.1371/journal.pbio.3000610)
Supplement: S2 Table — (PDF) [file pbio.3000610.s016.pdf]

S2 Table. Inferred genome properties based on k-mer content

	Heterozygosity	Haploid length	Repeat Length	Unique Length	Read Error Rate
Minimum	0.0302	~249 Mb	105.6 Mb	143.2 Mb	0.00151
Maximum	0.0306	~249.2 Mb	105.7 Mb	143.4 Mb	0.00151
