# Supplementary material for: Whole-chromosome hitchhiking driven by a male-killing endosymbiont
Source: PLoS Biol. 2020 Feb 27;18(2):e3000610. doi: 10.1371/journal.pbio.3000610 (PMC7046192; doi:10.1371/journal.pbio.3000610)
Supplement: S3 Table — (PDF) [file pbio.3000610.s017.pdf]

S3 Table. Final *D. chrysippus* assembly statistics

Total number of scaffolds	726
Total length	322399471
Largest scaffold	5321723
GC (%)	30.52
N50	628022
N75	628022
L50	63
L75	141
Total number of scaffolds (>= 0 bp)	726
Total number of scaffolds (>= 1000 bp)	726
Total number of scaffolds (>= 5000 bp)	702
Total number of scaffolds (>= 10000 bp)	679
Total number of scaffolds (>= 25000 bp)	605
Total number of scaffolds (>= 50000 bp)	512
Total length (>= 0 bp)	322399471
Total length (>= 1000 bp)	322399471
Total length (>= 5000 bp)	322335789
Total length (>= 10000 bp)	322161153
Total length (>= 25000 bp)	320856651
Total length (>= 50000 bp)	317312449
Number of N's per 100 kbp	1113.12
