# Supplementary material for: Whole-chromosome hitchhiking driven by a male-killing endosymbiont
Source: PLoS Biol. 2020 Feb 27;18(2):e3000610. doi: 10.1371/journal.pbio.3000610 (PMC7046192; doi:10.1371/journal.pbio.3000610)
Supplement: S4 Table — CEG, Core Eukaryotic Genes; CEGMA, Core Eukaryotic Genes Mapping Approach. (PDF) [file pbio.3000610.s018.pdf]

S4 Table. Summarized results of the CEGMA analysis based on 248 CEGs

Groups	Number of Proteins	Completeness	Total	Average	Percentage of Orthologs
Complete	192	77.42	224	1.17	13.02
Group 1	52	78.89	58	1.12	11.54
Group 2	42	75.00	48	1.14	9.52
Group 3	45	73.77	52	1.16	13.33
Group 4	53	81.54	66	1.25	16.98
Partial	224	90.32	304	1.36	28.57
Group 1	60	90.91	75	1.25	23.33
Group 2	47	83.93	67	1.43	31.91
Group 3	55	90.16	75	1.36	29.09
Group 4	62	95.38	87	1.40	30.65
