# Supplementary material for: Whole-chromosome hitchhiking driven by a male-killing endosymbiont
Source: PLoS Biol. 2020 Feb 27;18(2):e3000610. doi: 10.1371/journal.pbio.3000610 (PMC7046192; doi:10.1371/journal.pbio.3000610)
Supplement: S5 Table — (PDF) [file pbio.3000610.s019.pdf]

S5 Table. BUSCO statistics for 3 clades

Clade	Total BUSCO	Complete BUSCO	Single copy	Duplicated	Fragmented	Missing
Eukaryota	303	279	273	6	4	20
Arthropoda	1066	1002	994	8	14	50
Insecta	1658	1563	1547	16	17	78
