# Supplementary material for: Whole-chromosome hitchhiking driven by a male-killing endosymbiont
Source: PLoS Biol. 2020 Feb 27;18(2):e3000610. doi: 10.1371/journal.pbio.3000610 (PMC7046192; doi:10.1371/journal.pbio.3000610)
Supplement: S6 Table — (PDF) [file pbio.3000610.s020.pdf]

S6 Table. Summary of gene features in *D. chrysippus* genome

Total sequence length	323361855
Number of genes	16654
Number of mRNAs	18300
Number of exons	98865
Number of introns	80565
Number of CDS	15682
Total gene length	63146110
Total mRNA length	66260632
Total exon length	20536415
Total intron length	45885347
Total CDS length	20328882
mean gene length	3792
mean mRNA length	3621
mean exon length	208
mean intron length	570
mean CDS length	1296
% of genome covered by genes	19.5%
% of genome covered by CDS	6.3%
