# Supplementary material for: Whole-chromosome hitchhiking driven by a male-killing endosymbiont
Source: PLoS Biol. 2020 Feb 27;18(2):e3000610. doi: 10.1371/journal.pbio.3000610 (PMC7046192; doi:10.1371/journal.pbio.3000610)
Supplement: S7 Table — (PDF) [file pbio.3000610.s021.pdf]

S7 Table. Orthogroups summary statistics

Number of genes	74925
Number of genes in orthogroups	66709
Number of unassigned genes	8216
Percentage of genes in orthogroups	89.0
Percentage of unassigned genes	11.0
Number of orthogroups	12738
Number of species-specific orthogroups	39
Number of genes in species-specific orthogroups	173
Percentage of genes in species-specific orthogroups	0.2
Mean orthogroup size	5.2
Median orthogroup size	5.0
G50 (assigned genes)	5
G50 (all genes)	5
O50 (assigned genes)	4393
O50 (all genes)	5214
Number of orthogroups with all species present	7355
Number of single-copy orthogroups	4858
