# Supplementary material for: Whole-chromosome hitchhiking driven by a male-killing endosymbiont
Source: PLoS Biol. 2020 Feb 27;18(2):e3000610. doi: 10.1371/journal.pbio.3000610 (PMC7046192; doi:10.1371/journal.pbio.3000610)
Supplement: S8 Table — (PDF) [file pbio.3000610.s022.pdf]

## S8 Table. Distribution of orthogroups in different species

| Species | #genes | #genes in OG | #OG containing species |
|---|---|---|---|
| *Bombyx mori* | 14623 | 12653 (86.5%) | 10211 (80.2%) |
| *Danaus chrysippus* | 15675 | 14895 (95%) | 11343 (89%) |
| *Danaus plexippus* | 15130 | 14241 (94.1%) | 12015 (94.3%) |
| *Heliconius melpomene* | 12829 | 11954 (93.2%) | 10034 (78.8%) |
| *Melitaea cincia* | 16668 | 12966 (77.8%) | 10379 (81.5%) |
