# Supplementary material for: Whole-chromosome hitchhiking driven by a male-killing endosymbiont
Source: PLoS Biol. 2020 Feb 27;18(2):e3000610. doi: 10.1371/journal.pbio.3000610 (PMC7046192; doi:10.1371/journal.pbio.3000610)
Supplement: S9 Table — (PDF) [file pbio.3000610.s023.pdf]

S9 Table. Sample information for population genomic analyses

ID	Taxon / Population	Sex	Location	Lat.	Long.	Total Gb	Mean Depth	GanBank Accession
RF.W001	*Danaus chrysippus dorippus*	F	Watamu, Kenya	-3.33	40.02	11.49*	35.94*	SAMN08826813
RF.W002	*Danaus chrysippus dorippus*	M	Watamu, Kenya	-3.33	40.02	10.38*	32.45*	SAMN08826814
SM15.W61	*Danaus chrysippus dorippus*	M	Watamu, Kenya	-3.33	40.02	7.47	23.37	SAMEA6427843
SM15.W66	*Danaus chrysippus dorippus*	M	Watamu, Kenya	-3.33	40.02	7.15	22.35	SAMEA6427844
SM15.W69	*Danaus chrysippus dorippus*	M	Watamu, Kenya	-3.33	40.02	6.95	21.73	SAMEA6427845
SM15.W72	*Danaus chrysippus dorippus*	M	Watamu, Kenya	-3.33	40.02	9.82	30.72	SAMEA6427846
SM15.W74	*Danaus chrysippus dorippus*	M	Watamu, Kenya	-3.33	40.02	7.86	24.57	SAMEA6427847
RV12.N317	*Danaus chrysippus chrysippus*	F	El Haouareb, Tunisia	35.549	9.754	7.54	23.59	SAMEA6427848
SM17.P01	*Danaus chrysippus chrysippus*	F	Philippines (via Stratford Butterfly Farm)	?	?	8.15	25.48	SAMEA6427849
SM16.N01	*Danaus chrysippus alcippus*	F	Kanyang, Nigeria	6.24	8.97	8.56	26.76	SAMEA6427850
SM16.N04	*Danaus chrysippus alcippus*	F	Kanyang, Nigeria	6.24	8.97	7.73	24.17	SAMEA6427851
SM16.N05	*Danaus chrysippus alcippus*	M	Kanyang, Nigeria	6.24	8.97	7.18	22.45	SAMEA6427852
SM16.N06	*Danaus chrysippus alcippus*	M	Kanyang, Nigeria	6.24	8.97	6.84	21.4	SAMEA6427853
SM16.N20	*Danaus chrysippus alcippus*	M	Kanyang, Nigeria	6.24	8.97	8.11	25.36	SAMEA6427854
SM16.N37	*Danaus chrysippus alcippus*	M	Kanyang, Nigeria	6.24	8.97	8.11	25.37	SAMEA6427855
SM16.S03	*Danaus chrysippus orientis*	M	Magaliesberg, South Africa	-26.02	27.51	8.39	26.23	SAMEA6427856
SM16.S06	*Danaus chrysippus orientis*	M	Magaliesberg, South Africa	-26.02	27.51	7.12	22.28	SAMEA6427857
SM16.S11	*Danaus chrysippus orientis*	M	Magaliesberg, South Africa	-26.02	27.51	7.91	24.72	SAMEA6427858
SM16.S12	*Danaus chrysippus orientis*	M	Magaliesberg, South Africa	-26.02	27.51	7.32	22.9	SAMEA6427859
SM16.S14	*Danaus chrysippus orientis*	M	Magaliesberg, South Africa	-26.02	27.51	7.58	23.7	SAMEA6427860
SM16.S15	*Danaus chrysippus orientis*	M	Magaliesberg, South Africa	-26.02	27.51	7.69	24.04	SAMEA6427861
SM17.S01	*Danaus chrysippus orientis*	M	Magaliesberg, South Africa	-26.02	27.51	8.30	25.97	SAMEA6427862

RF.K001	contact zone	F	Nairobi, Kenya	-1.39	36.82	11.70*	36.59*	SAMN08826815
SM16.K622	contact zone	M	Nairobi, Kenya	-1.39	36.82	7.72	24.15	SAMEA6427863
SM16.K634	contact zone	F	Nairobi, Kenya	-1.39	36.82	7.72	24.13	SAMEA6427864
SM16.K641	contact zone	F	Nairobi, Kenya	-1.39	36.82	9.12	28.53	SAMEA6427865
SM16.K567	contact zone	F	Nairobi, Kenya	-1.39	36.82	7.30	22.83	SAMEA6427866
SM16.K570	contact zone	F	Nairobi, Kenya	-1.39	36.82	6.74	21.07	SAMEA6427867
SM16.K571	contact zone	F	Nairobi, Kenya	-1.39	36.82	7.64	23.89	SAMEA6427868
SM16.K577	contact zone	F	Nairobi, Kenya	-1.39	36.82	8.20	25.63	SAMEA6427869
SM16.K580	contact zone	F	Nairobi, Kenya	-1.39	36.82	8.28	25.9	SAMEA6427870
SM16.K584	contact zone	F	Nairobi, Kenya	-1.39	36.82	8.15	25.5	SAMEA6427871
SM16.K618	contact zone	M	Nairobi, Kenya	-1.39	36.82	8.61	26.94	SAMEA6427872
SM16.K626	contact zone	M	Nairobi, Kenya	-1.39	36.82	7.63	23.87	SAMEA6427873
SM16.K627	contact zone	F	Nairobi, Kenya	-1.39	36.82	6.67	20.87	SAMEA6427874
SM16.K628	contact zone	F	Nairobi, Kenya	-1.39	36.82	7.85	24.55	SAMEA6427875
SM16.K630	contact zone	F	Nairobi, Kenya	-1.39	36.82	7.20	22.53	SAMEA6427876
SM16.K631	contact zone	F	Nairobi, Kenya	-1.39	36.82	7.61	23.8	SAMEA6427877
SM16.K633	contact zone	F	Nairobi, Kenya	-1.39	36.82	7.59	23.75	SAMEA6427878
SM16.K620	contact zone	M	Nairobi, Kenya	-1.39	36.82	8.79	27.49	SAMEA6427879
SM16.K585	contact zone	F	Nairobi, Kenya	-1.39	36.82	7.38	23.07	SAMEA6427880
SM17.X01	cured line	F	Stock	NA	NA	8.32	26.02	SAMEA6427881
pet.A113	*Danaus petilia*	M	Australia	?	?	7.37	23.05	SAMN02996390
gil.C30	*Danaus gilippus*	F	Costa Rica	?	?	8.00	25.02	SAMN02996393
gil.T01	*Danaus gilippus*	M	Texas, USA	?	?	15.41	48.19	SAMN02996395

* indicates sequence content after down-sampling to reduce file size and processing time.
