# Supplementary material for: Whole-chromosome hitchhiking driven by a male-killing endosymbiont
Source: PLoS Biol. 2020 Feb 27;18(2):e3000610. doi: 10.1371/journal.pbio.3000610 (PMC7046192; doi:10.1371/journal.pbio.3000610)
Supplement: S10 Table — Shading indicates the best candidate gene(s) with the most nearby associated SNPs. (PDF) [file pbio.3000610.s024.pdf]

S10 Table. Closest genes to SNPs most strongly associated with colour pattern traits. Shading indicates the best candidate gene(s) with the most nearby associated SNPs.

Trait Association	Gene	Nearby SNPs	Description	e-Value
A	gene015410	3	transmembrane and TPR repeat-containing protein CG4341-like	9.58843E-151
A	gene015308	2	pheromone biosynthesis-activating neuropeptide receptor isoform B	1.0891E-33
A	gene003441	1	putative Calnexin	0
A	gene003471	1	transcriptional enhancer factor TEF-1 like protein	0
A	gene005973	1	serine protease 5	1.9592E-154
A	gene006029	1	hypothetical protein KGM_208582	0
A	gene006044	1	REPAT32 protein	1.8188E-28
A	gene006082	1	uncharacterized protein LOC113497409	1.74006E-133
A	gene013049	1	hypothetical protein KGM_211895	2.11066E-34
A	gene013615	1	splicing factor proline- and glutamine-rich	0
A	gene015600	1	cuticle protein	4.02653E-118
B	gene001666	4	yellow	0
B	gene001484	1	hypothetical protein KGM_206584	1.96903E-104
B	gene003564	1	amino acid transporter	0
B	gene005522	1	serine protease inhibitor 33 precursor	0
B	gene009383	1	UDP-glycosyltransferase UGT33J1	0
B	gene009488	1	Ecdysteroid UDP-glucosyltransferase	4.26885E-143
B	gene009489	1	UDP-glycosyltransferase UGT33F1	0
B	gene009492	1	UDP-glycosyltransferase UGT33F1	0
B	gene009493	1	UDP-glycosyltransferase UGT33F1	0
B	gene009511	1	uncharacterized protein LOC112045604	0
B	gene013199	1	hypothetical protein KGM_206574	5.47109E-67
B	gene013913	1	putative structural maintenance of chromosomes 5 smc5	0
B	gene013915	1	hemolymph proteinase 24	1.29126E-164
C	gene000403	2	low-density lipoprotein receptor-related protein 6 (arrow)	0
C	gene001633	2	hemolin-interacting protein	1.23689E-84
C	gene001631	1	hypothetical protein KGM_211925	1.56351E-117
C	gene003274	1	hypothetical protein KGM_200756	1.70394E-09
C	gene003458	1	ribosomal protein L27A	4.35329E-76
C	gene003477	1	hypothetical protein KGM_205676	0
C	gene003558	1	tetraspanin-9 like protein	5.36757E-139
C	gene011502	1	trypsin protein precursor	1.43537E-173
C	gene011692	1	uncharacterized protein LOC113397363	2.12478E-66
C	gene011696	1	hypothetical protein KGM_200711	0
C	gene013034	1	orexin receptor type 1-like	6.6138E-138
