# Supplementary material for: Whole-chromosome hitchhiking driven by a male-killing endosymbiont
Source: PLoS Biol. 2020 Feb 27;18(2):e3000610. doi: 10.1371/journal.pbio.3000610 (PMC7046192; doi:10.1371/journal.pbio.3000610)
Supplement: S11 Table — (PDF) [file pbio.3000610.s025.pdf]

S11 Table. Details of genotyping assays

Assay Details	Target	Forward primer	Reverse primer
“sc11_PCR_RFLP_2” (Fragment length polymorphisms without cleavage)	sc0000011 571266-571486 (chr15)	AAGAGTTTTAGCGCCGTAAG	CAACTCCTTGTCGTAATGATGA
“sc120_PCR_RFLP_2” RFLP with enzyme NdeI	sc0000120 387153-387517 (chr15)	CTTTCGCAAAGCCAAGGGAC	TAATCGAGGCGACCACAGTG
“COI2” Sequencing and RFLP with enzyme BsmI	Cytochrome Oxidase Subunit I (COI), Mitochondirial	GCTTAAACTCAGCCATTTTATTAGCG	TGGATCTCCTCCTCCAGCAG
“GDP1” PCR assay for *Spiroplasma* infection	*Spiroplasma* glycero-phosphoryl diester phospho-diesterase (GDP) gene	GAAAATTTGCCAAGCAGTAGAG	AACTACGGAAATTGAAGGATGC
