# Supplementary material for: Whole-chromosome hitchhiking driven by a male-killing endosymbiont
Source: PLoS Biol. 2020 Feb 27;18(2):e3000610. doi: 10.1371/journal.pbio.3000610 (PMC7046192; doi:10.1371/journal.pbio.3000610)
Supplement: S12 Table — Screening for mitochondrial type was either through direct sequencing or PCR RFLP for a diagnostic SNP in the COI amplicon. Screening for infection status was either based on resequencing data (see S11 Fig) or by PCR amplification of the Spiroplasma GDP gene. COI, Cytochrome Oxidase Subunit I; GDP, glycerophosphoryl diester phosphodiesterase; RFLP, restriction fragment length polymorphism. (PDF) [file pbio.3000610.s026.pdf]

S12 Table. Mitochondrial haplotype and infection status of 158 samples screened
Screening for mitochondrial type was either through direct sequencing or PCR-RFLP for a diagnostic SNP in the COI amplicon. Screening for infection status was either based on resequecning data (see S11 Fig) or by PCR amplification of the *Spiroplasma* GDP1 gene.

ID	Sex	wild/reared	latitude	longitude	K lineage SNP	Infection Status (GDP PCR)	PCR or Whole Genome Sequencing	COI Seqenced
SM16.S03	M	wild	-26.02	27.51	0	0	WGS	yes
SM16.S06	M	wild	-26.02	27.51	0	0	WGS	yes
SM16.S11	M	wild	-26.02	27.51	0	0	WGS	yes
SM16.S12	M	wild	-26.02	27.51	0	0	WGS	yes
SM16.S14	M	wild	-26.02	27.51	0	0	WGS	yes
SM16.S15	M	wild	-26.02	27.51	0	0	WGS	yes
SM17.S01	M	wild	-26.02	27.51	0	0	WGS	yes
SM17.H01	F	wild	-5.7175	-15.9249	0	0	PCR	no
SM17.H02	F	wild	-5.7175	-15.9249	0	0	PCR	no
SM17.H03	F	wild	-5.7175	-15.9249	0	0	PCR	no
SM17.H04	M	wild	-5.7175	-15.9249	0	0	PCR	no
DS06.A07	F	wild	-5.1	38.63	0	0	PCR	no
DS06.A12	F	wild	-5.1	38.63	0	0	PCR	no
DS06.A13	F	wild	-5.1	38.63	0	0	PCR	no
DS06.A14	F	wild	-5.1	38.63	0	0	PCR	no
DS06.A16	F	wild	-5.1	38.63	0	0	PCR	no
DS06.A17	F	wild	-5.1	38.63	0	0	PCR	no
DS06.A18	F	wild	-5.1	38.63	0	0	PCR	no
DS06.A19	F	wild	-5.1	38.63	0	0	PCR	no
RF.W001	F	wild	-3.33	40.02	0	0	WGS	yes
RF.W002	M	wild	-3.33	40.02	0	0	WGS	yes
SM15.W61	M	wild	-3.33	40.02	0	0	WGS	yes
SM15.W66	M	wild	-3.33	40.02	0	0	WGS	yes
SM15.W69	M	wild	-3.33	40.02	0	0	WGS	yes
SM15.W72	M	wild	-3.33	40.02	0	0	WGS	yes
SM15.W74	M	wild	-3.33	40.02	0	0	WGS	yes
SM16.W73	M	wild	-3.33	40.02	0	0	PCR	no
SM17.W01	F	wild	-3.33	40.02	0	0	PCR	no
SM17.R20	F	wild	-2.22	30.12	0	0	PCR	no
SM17.R21	M	wild	-2.22	30.12	0	0	PCR	no
SM17.R22	M	wild	-2.22	30.12	0	0	PCR	no

SM17.R23	F	wild	-2.22	30.12	1	1	PCR	yes
SM18.R009	M	wild	-2.22	30.12	1	0	PCR	no
SM18.R010	F	wild	-2.22	30.12	0	0	PCR	no
SM18.R011	F	wild	-2.22	30.12	0	0	PCR	no
SM18.R012	M	wild	-2.22	30.12	1	0	PCR	yes
SM18.M002	M	wild	-1.9	36.048	0	0	PCR	no
SM18.M003	F	wild	-1.9	36.048	0	0	PCR	no
RF.K001	F	wild	-1.39	36.82	1	1	WGS	yes
SM16.K567	F	wild	-1.39	36.82	1	1	WGS & PCR	yes
SM16.K570	F	wild	-1.39	36.82	1	1	WGS	yes
SM16.K571	F	wild	-1.39	36.82	1	1	WGS & PCR	yes
SM16.K577	F	wild	-1.39	36.82	1	1	WGS & PCR	yes
SM16.K580	F	wild	-1.39	36.82	1	1	WGS & PCR	yes
SM16.K583	F	wild	-1.39	36.82	1	1	PCR	no
SM16.K584	F	wild	-1.39	36.82	1	1	WGS & PCR	yes
SM16.K585	F	wild	-1.39	36.82	1	1	WGS & PCR	yes
SM16.K618	M	wild	-1.39	36.82	0	0	WGS & PCR	yes
SM16.K619	M	wild	-1.39	36.82	0	0	PCR	no
SM16.K620	M	wild	-1.39	36.82	1	0	WGS & PCR	yes
SM16.K622	M	wild	-1.39	36.82	0	0	WGS & PCR	yes
SM16.K624	M	wild	-1.39	36.82	1	1	PCR	no
SM16.K625	M	wild	-1.39	36.82	1	0	PCR	no
SM16.K626	M	wild	-1.39	36.82	0	0	WGS & PCR	yes
SM16.K627	F	wild	-1.39	36.82	1	1	WGS & PCR	yes
SM16.K628	F	wild	-1.39	36.82	1	1	WGS & PCR	yes
SM16.K630	F	wild	-1.39	36.82	1	1	WGS & PCR	yes
SM16.K631	F	wild	-1.39	36.82	1	1	WGS	yes
SM16.K633	F	wild	-1.39	36.82	1	1	WGS & PCR	yes
SM16.K634	F	wild	-1.39	36.82	1	1	WGS & PCR	yes
SM16.K641	F	wild	-1.39	36.82	1	1	WGS & PCR	yes
SM16.K700	F	wild	-1.39	36.82	1	1	PCR	no
ABRI.2018.3087	F	wild	-1.304	36.693	1	0	PCR	no
ABRI.2018.3088	M	wild	-1.304	36.693	1	1	PCR	no
ABRI.2018.3089	F	wild	-1.304	36.693	1	1	PCR	no
ABRI.2018.3090	F	wild	-1.304	36.693	0	0	PCR	yes
ABRI.2018.3091	F	wild	-1.304	36.693	1	1	PCR	no
ABRI.2018.3092	F	wild	-1.304	36.693	1	1	PCR	no
ABRI.2018.3093	F	wild	-1.304	36.693	1	1	PCR	yes
ABRI.2018.3094	F	wild	-1.304	36.693	1	0	PCR	yes

ABRI.2018.3095	F	wild	-1.304	36.693	1	0	PCR	no
ABRI.2018.3096	F	wild	-1.304	36.693	0	0	PCR	no
ABRI.2018.3097	F	wild	-1.304	36.693	1	1	PCR	no
ABRI.2018.3098	F	wild	-1.304	36.693	1	0	PCR	no
ABRI.2018.3099	F	wild	-1.304	36.693	1	1	PCR	no
ABRI.2018.3100	F	wild	-1.304	36.693	1	1	PCR	no
SM15.K01	F	wild	-1.304	36.693	0	0	PCR	yes
SM15.K02	M	wild	-1.304	36.693	0	0	PCR	yes
SM15.K03	F	wild	-1.304	36.693	0	0	PCR	yes
SM15.K04	M	wild	-1.304	36.693	0	0	PCR	yes
SM15.K05	F	wild	-1.304	36.693	0	0	PCR	yes
SM15.K06	M	wild	-1.304	36.693	0	0	PCR	no
SM15.K07	F	wild	-1.304	36.693	0	0	PCR	no
SM15.K08	M	wild	-1.304	36.693	0	0	PCR	no
SM15.K09	M	wild	-1.304	36.693	0	0	PCR	no
SM15.K10	M	wild	-1.304	36.693	0	0	PCR	no
SM15.K21	F	wild	-1.304	36.693	1	1	PCR	yes
SM16.L01	M	wild	0.302	36.908	0	0	PCR	no
SM16.L02	F	wild	0.302	36.908	1	1	PCR	yes
SM16.L03	F	wild	0.302	36.908	1	1	PCR	yes
SM16.L04	F	wild	0.302	36.908	0	0	PCR	no
SM16.L05	F	wild	0.302	36.908	1	1	PCR	no
SM16.L06	F	wild	0.302	36.908	0	0	PCR	no
SM16.L07	F	wild	0.302	36.908	1	1	PCR	no
SM16.L08	F	wild	0.302	36.908	1	1	PCR	no
SM16.L09	M	wild	0.302	36.908	0	0	PCR	yes
SM16.L10	M	wild	0.302	36.908	0	0	PCR	no
SM16.L11	F	wild	0.302	36.908	0	0	PCR	yes
SM16.L12	F	wild	0.302	36.908	0	0	PCR	no
SM16.L13	F	wild	0.302	36.908	0	0	PCR	no
SM16.L14	F	wild	0.302	36.908	0	0	PCR	no
SM16.L15	M	wild	0.302	36.908	0	0	PCR	no
SM16.L16	M	wild	0.302	36.908	0	0	PCR	no
SM16.L17	F	wild	0.302	36.908	0	0	PCR	no
SM16.L18	F	wild	0.302	36.908	0	0	PCR	no
SM16.L19	F	wild	0.302	36.908	0	0	PCR	no
SM16.L20	F	wild	0.302	36.908	0	0	PCR	no
SM16.L21	F	wild	0.302	36.908	0	0	PCR	no
SM16.L22	M	wild	0.302	36.908	0	0	PCR	yes

SM16.L23	M	wild	0.302	36.908	0	0	PCR	no
SM16.L24	M	wild	0.302	36.908	0	0	PCR	no
SM16.L25	M	wild	0.302	36.908	0	0	PCR	no
SM16.L26	M	wild	0.302	36.908	0	0	PCR	no
SM16.L27	M	wild	0.302	36.908	0	0	PCR	no
SM16.L28	M	wild	0.302	36.908	0	0	PCR	no
SM16.L29	F	wild	0.302	36.908	0	0	PCR	no
SM16.L30	F	wild	0.302	36.908	0	0	PCR	no
SM16.L31	M	wild	0.302	36.908	0	0	PCR	no
SM16.L32	F	wild	0.302	36.908	0	0	PCR	no
SM16.L33	F	wild	0.302	36.908	0	0	PCR	no
SM16.L34	F	wild	0.302	36.908	0	0	PCR	yes
SM16.L35	F	wild	0.302	36.908	0	0	PCR	no
SM16.L36	M	wild	0.302	36.908	0	0	PCR	no
SM16.L37	F	wild	0.302	36.908	0	0	PCR	no
SM16.L38	F	wild	0.302	36.908	0	0	PCR	no
SM16.L39	F	wild	0.302	36.908	0	0	PCR	no
SM16.L40	M	wild	0.302	36.908	0	0	PCR	no
SM16.L41	M	wild	0.302	36.908	0	0	PCR	no
SM16.L42	F	wild	0.302	36.908	0	0	PCR	no
SM16.M03	F	wild	0.302	36.908	0	0	PCR	no
SM16.N01	F	wild	6.24	8.97	0	0	WGS	yes
SM16.N04	F	wild	6.24	8.97	0	0	WGS & PCR	yes
SM16.N05	M	wild	6.24	8.97	0	0	WGS	yes
SM16.N06	M	wild	6.24	8.97	0	0	WGS	yes
SM16.N20	M	wild	6.24	8.97	0	0	WGS	yes
SM16.N37	M	wild	6.24	8.97	0	0	WGS	yes
DS98.324	F	wild	17.12	54.1	0	0	PCR	no
SM17.F01	F	wild	28.331	-13.923	0	0	PCR	no
SM17.F02	F	wild	28.331	-13.923	0	0	PCR	no
SM17.F03	F	wild	28.331	-13.923	0	0	PCR	no
SM17.F04	F	wild	28.331	-13.923	0	0	PCR	no
RV12.N314	U	wild	35.549	9.754	0	0	PCR	yes
RV12.N315	U	wild	35.549	9.754	0	0	PCR	no
RV12.N317	F	wild	35.549	9.754	0	0	WGS	yes
18MX101	F	reared	NA	NA	1	1	PCR	yes
18MX102	F	reared	NA	NA	1	1	PCR	no
FJ97.1011	F	reared	NA	NA	1	1	PCR	no
FJ97.1017	F	reared	NA	NA	1	1	PCR	no

FJ97.1026	F	reared	NA	NA	1	1	PCR	no
FJ97.1041	F	reared	NA	NA	1	1	PCR	yes
FJ97.1283	F	reared	NA	NA	0	0	PCR	yes
FJ97.1341	F	reared	NA	NA	0	0	PCR	no
FJ97.466	F	reared	NA	NA	1	1	PCR	no
FJ97.470	F	reared	NA	NA	1	1	PCR	no
FJ97.491	F	reared	NA	NA	1	1	PCR	yes
FJ97.633	F	reared	NA	NA	1	1	PCR	no
SM17.P01	F	reared	NA	NA	0	0	WGS	yes
SM17.X01	F	reared	NA	NA	1	0	WGS	yes
