# Supplementary material for: Whole-chromosome hitchhiking driven by a male-killing endosymbiont
Source: PLoS Biol. 2020 Feb 27;18(2):e3000610. doi: 10.1371/journal.pbio.3000610 (PMC7046192; doi:10.1371/journal.pbio.3000610)
Supplement: S1 Text — (PDF) [file pbio.3000610.s027.pdf]

S1 Text: Supplementary Methods

Reference genome sequencing and assembly

An adult female from the Nairobi area (RF.K001) was used to generate short insert libraries (500 bp) for paired end sequencing, and another female from the same brood was used to generate mate-pair libraries (insert sizes of 2 kb, 4.2 kb, 8.5 kb, 9.5 kb and 11.5 kb). Sequencing was performed using Illumina HiSeq 2500 technology with a read length of 250 bp. In total more than half a billion reads (336,807,664 x 2 = 673,615,328) containing more than 892 Gb (892,999,615,400 bp) of raw sequence generated from these two sisters (S1 Table).

Read files were quality checked using FastQC v0.10.1 [1]. Reads from small insert libraries were pre-processed with Trimmomatic v0.33 [2] to remove external adapter contamination as well as a minimum length threshold of 100 bp to discard false mate pair reads (ILLUMINACLIP::2:7:7 MINLEN:100).

All adapter-trimmed reads were checked for possible contamination using FastqScreen v0.5.2 [3] with libraries from human (*Homo sapiens* GRCh38), mouse (*Mus musculus* GRCm38), *E. coli* (U00096.3), Enterobacteria phage phiX174 (NC_001422.1), *Danaus chrysippus* mt genome as positive control (NC_026538.1) and simulated bacterial and viral databases from DeconSeq [4]. Most sequences could not be mapped to the provided libraries. Small fractions of reads could be mapped multiple times to multiple genomes which hint towards similar repeats in the provided libraries.

Genome size and overall genome characteristics (total and haploid genome length, percentage of repetitive content, and heterozygosity) were estimated from the k-mer profile using GenomeScope v1.0.0 [5]. This indicated high genome heterozygosity with a bi-modal k-mer distribution where the frequency of heterozygous k-mers (peak at 75x) were higher than homozygous k-mers (peak at ~150x). Inferred genome characteristics are given in S2 Table.

The k-mer profile of *Danaus chrysippus* suggested a high level of heterozygosity of about 3%. We therefore adopted a multi-step genome assembly pipeline designed to account for this heterozygosity. An initial draft assembly was generated by a single run of the SPAdes v3.8.1 [6] assembler using default parameters. For de-novo assembly, SPAdes selects multiple k-mer sizes, such as 21, 33, 55, 77, 99 and 127. Additional mate-pair data were incorporated to further scaffold the diploid assembly. We then used Redundans v0.12a [7] to generate a haplotype

resolved and scaffolded assembly. Scaffolding was performed using default parameters with all six (PE + MP) libraries in ascending order of average insert size according to the estimated ELF fraction. All sequences smaller than 500 bp were excluded from subsequent assembly steps. We iterated the Redundans runs multiple times to utilize reads with different orientations as a result of PE contamination in Nextera mate-pair data. Using the iterative approach we managed to increase the N50 of the assembly from 20kb to 204kb. However, the assembly size was still much higher than the 250 Mb (non-repetative fraction) estimated from the k-mer profile. We therefore used Haplomerger2 v3.4.0 [8,9], to further refine the scaffolds using mate-pair data with default parameters. Finally, a single scaffold of less than 1 kb was discarded and the mitochodrial scaffold and one contaminant scaffold were removed, to produce our final assembly. The final assembly N50 is 628 kb and the total assembly size is 322 Mb. Detailed characteristics are provided in S3 Table.

Repeat content analysis

A repeat library was created using dnaPipeTE 1.2 [10] and RepeatModeler 1.0.4 (http://www.repeatmasker.org/RepeatModeler/). For dnaPipeTE, mitochondrial and forward reads were excluded (as per the manual) from the complete trimmed raw read dataset, thereby producing an input file containing only reverse and unpaired reads from all libraries (168733627 reads / 42,183,406,750 bp). Firstly, dnaPipeTE was run on 30 coverage points ranging from 0.0006 to 0.8 with an estimated genome size of 400 MB. All parameters were set as default except the minimal contig length which was lowered from 200 to 50 bp. After analysing the N50 distribution from 30 coverage points, a coverage of 0.03 was selected as an optimal sampling coverage. 50 repetitions were performed with the selected optimal coverage and the same parameters as above. This resulted in 99,564 contigs with total length of 41,355,997 bp. The maximized N50 at 0.009 coverage was not viewed to be the optimal sampling coverage because the samples are very small and a huge variation in N50 was expected. Furthermore, filtering steps were introduced afterwards to discard non-repeats.

All trimmed reads were mapped un-paired against all contigs from dnaPipeTE using BWA mem v0.7.12 [11,12] with the options -t 80 -k 25 -a -y 26 -c 1000000000 and otherwise default settings. The coverage per position was calculated using samtools v1.3 [13] mpileup with options -A -C 50 -d 1000000 and otherwise default parameters. Contigs with a median coverage smaller than the 90% quantile (94x) of the per position coverage distribution were filtered out (3,876 out

of 99,564 contigs). The remaining 95,688 (40969029 bp total length) with sufficient coverage were used in the subsequent steps.

A repeat-protein free protein database was built from Swiss-Prot database [14] (accessed on March 30th 2017) and all repeat sequences from the Repbase database [15] (accessed on March 30th 2017). The repeat sequences were searched in the Swiss-Prot database via BlastX 2.3.0 [16,17] with an e-value cutoff of e10-11. Protein sequences with hits from repeat sequences containing at least 20 bp in one hit were removed to obtain a repeat-protein free protein database. The repeat families from dnaPipeTE runs were blasted (BlastX; e-value cutoff e10-11) against the repeat-protein free protein database. These sequences were removed from final repeat library containing 41261 sequences totalling a length of 17477008 and an N50 of 736 bp.

RepeatModeler was then executed with default parameters on the final genome assembly and on the contigs obtained from dnaPipeTE. The resulting fasta files containing the repeat families were concatenated into 936 sequences with a total length of 1,433,744 bp and an N50 of 2644 bp.

Assembly Completeness

The Core Eukaryotic Gene Mapping Algorithm, CEGMA v2.5.0 [18] and the Benchmarking Universal Single-Copy Orthologs, BUSCO v3.0.0 [19] were used to estimate the completeness of genome assembly and quality of gene annotation of the *D. chrysippus* final assembly. Results are presented in S4 Table and S5 Table. We ran BUSCO with bot the Arthropoda (n=1066) and Insecta (n=1058) core gene sets which identified 93% and 94% of the single copy genes searched, respectively. This suggest a high contiguity and completeness of our final assembly.

Genome Annotation

For annotation of the assembly we used the MAKER v2.31.8 pipeline [20,21] combined with MPICH2 (http://www.mpich.org/) in three iterations. Firstly, an Augustus species model was computed using the gene models obtained from BUSCO analysis. BUSCO was run on the assembly using the metazoan dataset together with the option --long and otherwise default parameters. From the CEGMA run on the assembly a SNAP [22] model was built using the script cegma2zff from the MAKER2 distribution and the SNAP scripts fathom (fathom genome.ann genome.dna -categorize 1000 && fathom -export 1000 -plus uni.ann uni.dna), forge (export.ann

export.dna) and hmm-assembler.pl. A Genemark [23] model was built using GeneMark-ES suite 4.32 from self-training (--ES) on the assembly. An initial MAKER2 run was executed using the *D. chrysippus* assembly, *D. chrysippus* ESTs (obtained from GenBank), and protein sequences from *Danaus plexippus* (as a protein homology evidence from an alternate closely related species) with options est2genome and protein2genome enabled. In the second MAKER2 run, the est2genome and protein2genome options were disabled and the *D. chrysippus* assembly was used as input, along with the Augustus species model, custom repeat library HMM models from SNAP and GeneMark. The minimum protein length was set to ten amino acids. After the first iteration, the gff file for the whole assembly was extracted using the MAKER2 gff3_merge, converted with maker2zff and a new HMM model was built for SNAP the same way as above. The Augustus species model was retrained locally by first converting with the SNAP script zff2gff3.pl (zff2gff3.pl genome.ann | perl -plne 's/\t(\S+)$/\t\.\t$1/') and second the autoAug.pl script from Augustus 3.2.2 [24]. The input for the autoAug.pl were the genome assembly, trained Augustus species model and the gff3 file created from the first MAKER2 iteration. Default parameters wre used, but with the options -v --useexisting. For the second MAKER2 iteration the SNAP HMM model was further improved using the results from first iteration. Additionally, the Augustus species model was also improved. At this point, the minimum protein length was raised to 30 amino acids. Thereafter, a retraining as above and a third MAKER2 run was performed. Functional annotation of the MAKER2 derived gene models were done using homology searches against BLAST nr, InterPro and KEGG databases. A summary of the annotation characteristics is provided in S6 Table.

Orthology analysis

Protein sets from *Danaus chrysippus*, three other butterfly species and a moth species were used to predict ortholog clusters using OrthoFinder v0.7.1 [25] with default parameters. Functional annotation of all protein sets with Gene Ontology (GO) terms was performed with BLAST2GO v4.1.0 [26] and InterProScan v5.0.0 [27] using default parameters. Results are presented in S7 Table S7 and S8 Table.

Pseudo-chromosomal assembly

We produced a pseudo-chromosomal assembly based on homology with the *Heliconius melpomene* Hmel2.5 genome, which is the most contiguous genome from the same family (Nymphalidae) currently available. Following [28], we numbered chromosomes based on the M.

cinxia genome [29]. *Danaus* have 30 chromosomes, which differ from the ancestral 31 (as found in *M. cinxia*) by a single fusion between chromosome 1 (the Z sex chromosome) and chromosome 21 [28]. However, *Heliconius melpomene* has 21 chromosomes due to 10 fusion events [29,30]. In order to assign *Danaus* chromosomes for each gene, we therefore first determined the tracts representing the ancestral 31 chromosomes in the ancestor of *Heliconius* by splitting at the fusion points identified by Davey et al. [30], re-numbering the chromosomes based on *M.* cinxia [29], and then fusing chromosomes 1 and 21 to effectively reorganise the *Heliconius* chromosomes into a *Danaus*-like karyotype..

We used BLASTp [16,17] to identify likely homology between the translated gene sets for the two species. Only hits with an e value < 10^{-20} and a minimum AA sequence identity of 50% were considered. We first screened for misassembled scaffolds by identifying all scaffolds with hits to two or more chromosomes, with the additional requirement that at least three genes (which had to represent at least 5% of genes on the scaffold) had hits to the lesser chromosome. All possible misassembled scaffolds were then inspected visually and likely breakpoints were identified in 80 scaffolds. The resulting modified assembly consisting of 945 scaffolds was used for all evolutionary analyses.

To produce a pseudo-chromosomal assembly, we discarded all scaffolds with fewer than four blast hits to a single chromosome except for those in which at least 75% of blast hits were to a single chromosome. In total 438 scaffolds, totalling 282 Mb (87% of the genome) could be assigned to chromosomes. Scaffolds were ordered based on the median position of their blast hits, and oriented based on the rank order of the start and end positions of all hits.

***Spiroplasma* genome**

Scaffolds corresponding to the genome of the *Spiroplasma* sp. endosymbiont were identified based on comparative read depth in suspected infected and uninfected samples (S10 Fig). Initially, only a single *Spiroplasma* scaffold was identified in the complete genome, but inspection of the second version of the genome (i.e. before running Haplomerger, see above) identified twelve scaffolds with high read depth in the infected sample and effectively zero read depth in the cured sample. Three scaffolds appeared to represent one or more *Spiroplasma* plasmids, as their read depth was considerably higher. One of the other nine scaffolds was evidently chimeric based on read depth, and this scaffold was broken manually. The resulting putative *Spiroplasma* genome has a total length of 1.7 Mb. We annotated the genome using

RAST [31,32], and confirmed its identity using BLAST [17] to available *Spiroplasma* genomes. All but the shortest scaffold had multiple hits to *Spiroplasma sp.* (e vlaue < 1e-10). The other two candidate plasmid scaffolds had hits to *Spiroplasma citri* plasmids. To test for infection in resequenced samples, reads were mapped to our *Spiroplasma* sp. genome using bwa mem v0.7.12 [11,12] and read depth was computed using Samtools v1.3 [13]. We also confirmed that other known male-killers *Wolbachia* and *Rickettsia* are not represented in our reference genome (from a member of an all female brood) using blast with published sequences (GenBank accessions AJ130716 and AJ269519).

Mitochondrial genome assemblies

We assembled the mitochondrial genomes for all 45 samples included in the study from Illumina short-read sequences using NOVOplasty [33]. A previously sequenced *D. chrysippus* mitochondrial genome (GenBank accession: NC_024532) was used as a seed. Although NOVOplasty generates de novo assemblies, a reference sequence can be provided to resolve duplicated regions. The same complete genome was therefore also provided as a reference. For all 42 *D. chrysippus* samples sequenced in this study, which were either 150 bp paired-end or 250 bp paired-end, we used a kmer size of 39, an insert size of 300 and an insert range of 1.8 (1.3 for repetitive regions). For the *D. petilia* and *D. gilippus* samples that were sequenced previously [34] (100 bp paired-end), we used a kmer size of 25, an insert size of 300 and an insert range of 2.4 (1.3 for repetitive regions).

Population sample resequencing and genotyping

DNA was extracted from thorax tissue using the Dneasy blood and tissue kit (Qiagen,). Paired end sequencing libraries were prepared using the Truseq Nano DNA HT Sample preparation Kit (Illumina USA) with the addition of individual indexes. Fragmentation to 350 bp was performed using the Covaris cracker. Fragments were prepared for Illumina sequencing via adapter ligation and further PCR amplification, followed by purification using the AMPure XP system (Beckman Coulter). Paired-end sequencing (150 bp) was performed using the HiSeq X (Illumina). All samples were sequenced to a mean depth of coverage 20x or greater.

Reads were mapped to the *D. chrysippus* reference assembly using Stampy [35] v1.0.31. BAM files were sorted using Samtools [13] v1.3 and PCR duplicate reads were removed using PicardTools MarkDuplicates v1.135 (https://broadinstitute.github.io/picard/). Genotyping was performed using GATK v3 HaplotypeCaller and GenotypeGVCFs [36,37] using default

parameters except that heterozygosity was set to 0.02. Genotyping was performed separately for each species. Genotype calls were required to have an individual depth ≥ 8x, and heterozygous and alternate allele calls were further required to have an individual genotype quality (GQ) ≥ 20.

References

1. Andrews S, Babraham Bioinformatics. FastQC: A quality control tool for high throughput sequence data. Manual. 2010. doi:citeulike-article-id:11583827

2. Bolger AM, Lohse M, Usadel B. Trimmomatic: a flexible trimmer for Illumina sequence data. Bioinformatics 2014;30: 2114–2120. doi:10.1093/bioinformatics/btu170

3. Wingett SW, Andrews S. FastQ Screen: A tool for multi-genome mapping and quality control. F1000Research 2018;7: 1338. doi:10.12688/f1000research.15931.2

4. Schmieder R, Edwards R. Fast Identification and Removal of Sequence Contamination from Genomic and Metagenomic Datasets. Rodriguez-Valera F, editor. PLoS One 2011;6: e17288. doi:10.1371/journal.pone.0017288

5. Vurture GW, Sedlazeck FJ, Nattestad M, Underwood CJ, Fang H, Gurtowski J, et al. GenomeScope: fast reference-free genome profiling from short reads. Berger B, editor. Bioinformatics 2017;33: 2202–2204. doi:10.1093/bioinformatics/btx153

6. Bankevich A, Nurk S, Antipov D, Gurevich AA, Dvorkin M, Kulikov AS, et al. SPAdes: A New Genome Assembly Algorithm and Its Applications to Single-Cell Sequencing. J Comput Biol 2012;19: 455–477. doi:10.1089/cmb.2012.0021

7. Pryszcz LP, Gabaldón T. Redundans: an assembly pipeline for highly heterozygous genomes. Nucleic Acids Res 2016;44: e113–e113. doi:10.1093/nar/gkw294

8. Huang S, Kang M, Xu A. HaploMerger2: rebuilding both haploid sub-assemblies from high-heterozygosity diploid genome assembly. Bioinformatics 2017;33: 2577–2579. doi:10.1093/bioinformatics/btx220

9. Huang S, Chen Z, Huang G, Yu T, Yang P, Li J, et al. HaploMerger: reconstructing allelic relationships for polymorphic diploid genome assemblies. Genome Res 2012;22: 1581–8. doi:10.1101/gr.133652.111

10. Goubert C, Modolo L, Vieira C, ValienteMoro C, Mavingui P, Boulesteix M. De Novo Assembly and Annotation of the Asian Tiger Mosquito (Aedes albopictus) Repeatome with dnaPipeTE from Raw Genomic Reads and Comparative Analysis with the Yellow Fever Mosquito (Aedes aegypti). Genome Biol Evol 2015;7: 1192–1205. doi:10.1093/gbe/evv050

11. Li H, Durbin R. Fast and accurate short read alignment with Burrows-Wheeler transform. Bioinformatics. 2009;25: 1754–1760. doi:10.1093/bioinformatics/btp324

12. Li H. Aligning sequence reads, clone sequences and assembly contigs with BWA-MEM. arXiv Prepr arXiv. 2013; doi:arXiv:1303.3997

13. Li H, Handsaker B, Wysoker A, Fennell T, Ruan J, Homer N, et al. The Sequence Alignment/Map format and SAMtools. Bioinformatics. 2009;25: 2078–9. doi:10.1093/bioinformatics/btp352

14. Boutet E, Lieberherr D, Tognolli M, Schneider M, Bairoch A. UniProtKB/Swiss-Prot. Plant Bioinformatics. 2007. pp. 89–112. doi:10.1007/978-1-59745-535-0_4

15. Bao Z, Eddy SR. Automated de novo identification of repeat sequence families in sequenced genomes. Genome Res. 2002;12: 1269–76. doi:10.1101/gr.88502

16. Camacho C, Coulouris G, Avagyan V, Ma N, Papadopoulos J, Bealer K, et al. BLAST+: architecture and applications. BMC Bioinformatics. 2009;10: 421. doi:10.1186/1471-2105-10-421

17. Altschul SF, Gish W, Miller W, Myers EW, Lipman DJ. Basic local alignment search tool. J Mol Biol. 1990;215: 403–10. doi:10.1016/S0022-2836(05)80360-2

18. Parra G, Bradnam K, Korf I. CEGMA: a pipeline to accurately annotate core genes in eukaryotic genomes. Bioinformatics. 2007;23: 1061–1067. doi:10.1093/bioinformatics/btm071

19. Simão FA, Waterhouse RM, Ioannidis P, Kriventseva E V., Zdobnov EM. BUSCO: assessing genome assembly and annotation completeness with single-copy orthologs. Bioinformatics. 2015;31: 3210–3212. doi:10.1093/bioinformatics/btv351

20. Cantarel BL, Korf I, Robb SMC, Parra G, Ross E, Moore B, et al. MAKER: an easy-to-use annotation pipeline designed for emerging model organism genomes. Genome Res. 2008;18: 188–96. doi:10.1101/gr.6743907

21. Holt C, Yandell M. MAKER2: an annotation pipeline and genome-database management tool for second-generation genome projects. BMC Bioinformatics. 2011;12: 491. doi:10.1186/1471-2105-12-491

22. Korf I. Gene finding in novel genomes. BMC Bioinformatics. 2004;5: 59. doi:10.1186/1471-2105-5-59

23. Lomsadze A, Ter-Hovhannisyan V, Chernoff YO, Borodovsky M. Gene identification in novel eukaryotic genomes by self-training algorithm. Nucleic Acids Res. 2005;33: 6494–6506. doi:10.1093/nar/gki937

24. Stanke M, Schöffmann O, Morgenstern B, Waack S. Gene prediction in eukaryotes with a generalized hidden Markov model that uses hints from external sources. BMC Bioinformatics. 2006;7: 62. doi:10.1186/1471-2105-7-62

25. Emms DM, Kelly S. OrthoFinder: solving fundamental biases in whole genome comparisons dramatically improves orthogroup inference accuracy. Genome Biol. 2015;16: 157. doi:10.1186/s13059-015-0721-2

26. Conesa A, Gotz S, Garcia-Gomez JM, Terol J, Talon M, Robles M. Blast2GO: a universal tool for annotation, visualization and analysis in functional genomics research. Bioinformatics. 2005;21: 3674–3676. doi:10.1093/bioinformatics/bti610

27. Jones P, Binns D, Chang H-Y, Fraser M, Li W, McAnulla C, et al. InterProScan 5: genome-scale protein function classification. Bioinformatics. 2014;30: 1236–1240. doi:10.1093/bioinformatics/btu031

28. Mongue AJ, Nguyen P, Voleníková A, Walters JR. Neo-sex chromosomes in the monarch butterfly, Danaus plexippus. G3. 2017;7: 3281–3294. doi:10.1534/g3.117.300187

29. Ahola V, Lehtonen R, Somervuo P, Salmela L, Koskinen P, Rastas P, et al. The Glanville fritillary genome retains an ancient karyotype and reveals selective chromosomal fusions in Lepidoptera. Nat Commun. 2014;5: 1–9. doi:10.1038/ncomms5737

30. Davey JW, Chouteau M, Barker SL, Maroja L, Baxter SW, Simpson F, et al. Major Improvements to the Heliconius melpomene Genome Assembly Used to Confirm 10 Chromosome Fusion Events in 6 Million Years of Butterfly Evolution. G3. 2016;6: 695–708. doi:10.1534/g3.115.023655

31. Aziz RK, Bartels D, Best AA, DeJongh M, Disz T, Edwards RA, et al. The RAST Server: Rapid Annotations using Subsystems Technology. BMC Genomics. 2008;9: 75. doi:10.1186/1471-2164-9-75

32. Overbeek R, Olson R, Pusch GD, Olsen GJ, Davis JJ, Disz T, et al. The SEED and the Rapid Annotation of microbial genomes using Subsystems Technology (RAST). Nucleic Acids Res. 2014;42: D206–D214. doi:10.1093/nar/gkt1226

33. Dierckxsens N, Mardulyn P, Smits G. NOVOPlasty: *de novo* assembly of organelle genomes from whole genome data. Nucleic Acids Res. 2016;45: gkw955. doi:10.1093/nar/gkw955

34. Zhan S, Zhang W, Niitepõld K, Hsu J, Haeger JF, Zalucki MP, et al. The genetics of monarch butterfly migration and warning colouration. Nature. 2014;514: 317–21. doi:10.1038/nature13812

35. Lunter G, Goodson M. Stampy: a statistical algorithm for sensitive and fast mapping of Illumina sequence reads. Genome Res. 2011;21: 936–9. doi:10.1101/gr.111120.110

36. DePristo M a, Banks E, Poplin R, Garimella K V, Maguire JR, Hartl C, et al. A framework for variation discovery and genotyping using next-generation DNA sequencing data. Nat Genet. 2011;43: 491–8. doi:10.1038/ng.806

37. Van der Auwera GA, Carneiro MO, Hartl C, Poplin R, del Angel G, Levy-Moonshine A, et al. From fastQ data to high-confidence variant calls: The genome analysis toolkit best practices pipeline. Curr Protoc Bioinforma. 2013;43: 11.10.1-11.10.33. doi:10.1002/0471250953.bi1110s43
