# Supplementary material for: Whole-chromosome hitchhiking driven by a male-killing endosymbiont
Source: PLoS Biol. 2020 Feb 27;18(2):e3000610. doi: 10.1371/journal.pbio.3000610 (PMC7046192; doi:10.1371/journal.pbio.3000610)
Supplement: Attachment — Submitted filename: Martin_et_al_20191112_responses_to_reviewers.pdf [file pbio.3000610.s028.pdf]

We thank the reviewers for their constructive comments on our manuscript. All three reviewers were positive and mainly requested increased clarification and elaboration, which we have now provided.

The following are the most significant changes in our revised version:

1. We have merged two figures into a single main figure of genetic differentiation and colour pattern association, and added two supplementary figures and a supplementary table on these results, including a new analysis of absolute genetic divergence (dxy).

2. We have added a bootstrapping analysis to allow statistical assessment of the excess nonsynonymous polymorphism on chromosome 15.

3. 1. We provide more extensive consideration of factors that might be driving (or limiting) spread of the neo-W and male killer, including meiotic drive and direct selection on colour pattern.

Our responses to all comments are provided below in blue.

****
REVIEWERS' COMMENTS:

Reviewer #1:

Summary

Martin et al. present a largely descriptive, but compelling, example of the recent spread of a neo-sex chromosome that is perfectly genetically linked to a male-killing endosymbiont. They show that the spread of these elements in this population has been recent and dramatic. Furthermore, the elements are perfectly linked, emphasizing the impact of sex-biased inheritance in amplifying the effects of a presumed selective sweep.

Overall the manuscript is extremely well written and the data are clearly presented. I have no major concerns with the methods presented and I am certain this will be of interest to the broad readership of PLOSbio.

Major

Overall I like the observation and generally think it's possible that spread of a selfish element has resulted in the spread of genetically linked mtDNA and the neo-W chromosome. However, it is also possible that selection has favored the neo-W either on its own or in addition to the spiroplasm infection. For example, it might be that the neo-sex chromosome experiences some amount of meiotic drive. In fact, if figure S6B reflects the average sex ratio associated with this chromosome, then the female-skew is significantly in excess of the expectation ($p = 0.047$, binomial test). In either event, the sex ratio of the cured line should be carefully examined and reported as an integral part of this work.

Furthermore, we might also expect meiotic drive of a linked-sex chromosome to evolve as a consequence of spiroplasm infection. I.e., this would mitigate the cost of male-killing by reducing

the base rate of males. In either case, the combination of meiotic drive and female-skewed sex ratios could then also be adaptive for both genetic elements.

I acknowledge these possibilities are complicated and clearly there is not sufficient data to confidently exclude any (though again, what is the sex ratio of the cured line?), but the text currently presents a simple view that the mtDNA and neo-sex chromosomes are hitchhiking on the spiroplasm infection. A much more nuanced discussion is therefore needed to explain this observation and clarify the possible evolutionary causes.

We thank the reviewer for this interesting suggestion that we did not give sufficient consideration in our previous submission. In the revised version, we consider it explicitly as follows:

"We cannot entirely rule out the possibility that the neo-W is contributing to this spread, or even driving it entirely, through direct selection or meiotic drive. In theory this is testable by examining broods that carry the neo-W but lack Spiroplasma. We raised 11 such broods in our cured line, and Smith [47] reported 10 natural broods that showed sex-linked colour pattern and no male killing. Across these 21 broods, totalling 528 adult offspring, 51% were female. This is far from significantly different from the null expectation of 50% (binomial test p=0.7). However, we note that in order to detect meiotic drive causing a 1% female bias with good power would require a far larger samples size of >15,000. Given the evidence from other work showing that male killers can spread rapidly as selfish elements [48], and the endosymbionts can convey other fitness benefits [49], this remains the most parsimonious explanation in this case."

Additionally, I understand that the coloration pattern is of historical significance, but I am unclear why so much of the work focuses on this. It seems like an interesting accident that there's an obvious phenotype associated with this neo-sex chromosome. In either event, I recommend reducing the section that focuses on coloration alleles since it is somewhat aside the main novelty of this work.

We have slightly reduced our description of the colour pattern associations, and also reduced this part to a single figure, with some results moved to a supplementary figure. However, another reviewer requested more extensive description of possible associated genes, which we now provided in a supplementary table. We think there is a good argument to retain a strong emphasis on colouration in our paper. Danaus chrysippus is best known for its amosematic colouration, and the polymorphism in the rift valley region has been of interest to authors other than us (e.g. Joron and Mallet 1998). Moreover, the theme of recombination suppression runs through both the colour pattern and neo-sex chromosome parts of our paper. We describe two distinct consequences of suppressed recombination: supergenes and hitchhiking, both of which are fundamental concepts in population genetics.

Minor

Lines 135-173. The weak association on chromosome 22 might also reflect errors in the scaffolding. The authors should acknowledge this alternative explanation, but need not discuss in detail since the overall pattern is obvious and this does not impact their primary conclusions.

There is good evidence that these association are indeed found on chromosome 22. They are spread across three large scaffolds that cover over 2 Mb and are all clearly associated with chrom. 22 by numerous blast hits. This can be seen in the revised version by comparing figures S1 and S2.

Lines 189-191. I find the case in Joron et al. (2011) fairly compelling, though perhaps the authors are leaning on the identification of specific candidate genes. Regardless, I think this statement of novelty is pretty weak and recommend removing it.

We have shortened and clarified our statement:

"*Our study is one of only a few cases in which it can be shown that alleles at distinct loci that each influence a component of a complex trait are maintained in LD by suppressed recombination [43,44].*"

To our knowledge, the above has not been proved in the case of the H. numata supergene.

Lines 214-215. Sample size is not mentioned in the main text for the cross experiment and this made me wonder how strong the results are. In figure S6 it is very clear that the sample size is sufficient to confidently support the authors' statement. I recommend including a short reference to the sample size and a P-value in the main text.
Sample sizes and chi-square test p-values have been added.

Line 250. The 100kb windows are non-independent due to linkage. I do not think a Wilcoxon test is the right choice for this. Though that's probably conservative here since the results support the null.

We agree that, if anything, non-independence among adjacent windows would inflate statistical significance in the test for enhanced heterozygosity in females carrying the neo-W. Since our p-value is 0.36, this just increases our confidence in the null result.

Moreover, we are confident that the effect on the test is minor. We have previously shown that there is limited serial correlation among 100 kb windows in a butterfly with a similarly large effective population size (Martin et al. 2016, Genetics).

Nonetheless, we repeated this analysis using only every second window, and the p-value changed from 0.36 to 0.42 (despite halving the sample size). We also performed another test in which we compared the average heterozygosity across the whole chromosome among individuals with (n=14) and without (n=5) the fusion. In this case p=0.96. If the reviewer and editor feel that it would be more suitable to use one of the latter two tests instead, we would be happy to do so, but we feel that describing all of these tests would be an unnecessary use of space since the null result is consistent across all of them.

LIne 254. "Linage" should be lineage.
Fixed, thank you.

Fig 5. I find the crossing lines in the figure to be slightly confusing as they imply non-concordance between the neo-W and symbiont genealogies, when in fact, there is no evidence for this. I recommend rearranging the tree so these can be displayed cleanly as parallel lines.
Thank you for pointing this out. We have corrected the figure.

Line 310. "unlinked" should probably be "physically unlinked".
Fixed, thank you.

The github repository appears to contain a large array of genomic analysis scripts. They are well documented, but it would be good to include the current versioning somewhere in the manuscript in case the scripts are changed at a later time.

This is a good suggestion. We have added versioning and indicated which version was used in the Methods when naming the scripts.

Reviewer #2:

Martin et al. have carried out a really interesting study that I think should be published. The authors provide pretty convincing support that a neo-W chromosome has hitchhiked to relatively high frequency via the spread of male-killing spiroplasma. This same (neo-W) chromosomal region contains two colour patterning loci, and due to male-killing the focal population consists of only infected females that carry identical colour patterning alleles. Their analyses provide some candidate genes for this colour pattern variation, although support is limited. Phylogenetic analyses suggest congruence of spiroplasma with the neo-W, supporting hitchhiking of the neo-W with spread of spiroplasma through the population. The lack of female recombination combined with male killing results in a population of only infected females that carry the same colour patterning allele. I have several comments that I hope the authors can address. I have put an asterisk on those comments that I think
are the most important, but generally I like this paper and think it contains some really exciting biology that readers will enjoy.

*Line 111: The authors should use dxy and compare to the fst results here since dxy makes interpretation much easier. Also, fst and population size are not related unless I misunderstand.

We thank the reviewer for this suggestion. While we understand the arguments in favour of absolute divergence measures like dxy over relative measures, absolute measures have their own problems caused by heterogeneity in levels of standing variation and substitution rates across the genome. We have therefore plotted dxy against within-population diversity (pi) in Figure S3. This shows that chromosomes 11 and 15 are indeed extreme outliers for divergence relative to within-population diversity, and that chr15 in particular includes some of the most divergent regions of the genome.

We agree that low Fst is not directly indicative of large Ne, although Fst between isolated populations will increase more slowly the larger their Ne due to reduced drift. We have re-worded this section to remove the suggestion that Fst directly implies anything about Ne:

"This low background level implies a large and nearly panmictic population across the continent. The effective population size appears to be very large, as average genome-wide diversity at putatively neutral 4-fold degenerate 3rd codon positions is 0.042, which is among the highest values reported for animals [33,34]"

Line 168: Be more precise here instead of stating "nearly perfect".

We have clarified this as follows:

"A cluster of SNPs most strongly associated with background colour (B locus) is found just upstream of the gene yellow, and a phylogenetic network for a 30 kb region around yellow groups individuals nearly perfectly by phenotype, although some individuals classed as heterozygous were intermingled with homozygotes (S5 Fig)."

*Lines 174-181: How many other genes are in this area, and how many of those have strongly associated SNPs? There are not many data to really implicate arrow here, and informing the reader

of other associated SNPs/genes would be useful. Perhaps a supplemental table for both the yellow and arrow regions that reports SNPs and genes would be useful.
This is a good suggestion. We have added a supplementary table naming all genes that were closet to one of the most strongly associated SNPs. This shows that *yellow* is the best candidate for the B locus, whereas the strong associations for the C locus are indeed less clearly indicative of a single gene. We think that *arrow* is still the best candidate due to its association with wnt signalling, but we do state in the paper that further verification is required.

*Line 192: Is obtaining long read (nanopore eg) difficult for some reason in this system? I ask because it would be incredibly useful here, for isolation of the neo W below, etc. In a few weeks it seems the authors could have these data, which would enable them to answer several of the outstanding questions. Unless this is particularly difficult in this system I would urge the authors to consider it.
We agree that it would be great to be able to reconstruct the structural rearrangements that differentiate the subspecies. However, we have had challenges generating useful long-read data for suitable samples, so this remains a focus of our ongoing work and is beyond the scope of this study. Importantly, the primary finding of our paper: that there is a supergene that is co-inherited with an endosymbiont, does not hinge on what rearrangements occurred to produce the supergene.

Line 197: How divergent is H. Melpomene? This would be useful to know when considering the synteny comparison.

Divergence between the ancestors of *Heliconius* and *Danaus* occurred around 90MYA (Wahlberg 2009). However, comparisons of synteny at this level of divergence have already been shown to be reliable to reconstruct *Danaus* chromosomes (Mongue et al. 2017). We now note this in the Methods. This is thanks to the generally stable macrosynteny of Lepidoptera, exemplified by the fact that most species retain the ancestral karyotype of n=31.

Importantly the finding that one cluster of genes on chr15 occurs in many copies in *D. chrysippus dorippus* was also validated (Figure S7) using read-depth analyses and building a gene family tree based on BLAST hits in two other butterfly genomes, including the much more closely related *Danaus plexippus* (which we acknowledge would have served as a better chromosomal reference had a chromosomal assembly been available).

*Line 210: Are your species infected with Wolbachia or other endosymbionts? Perhaps this is reported and I missed it, but knowing that spiroplasma is the only reproductive manipulator in the text here is crucial.

We had little reason to suspect other male killers as Jiggins et al. (2000, Parasitology) found a perfect association between *Spiroplasma* infection and male killing across over 80 broods.

We have now also performed BLAST searches using query sequences from known male killers *Wolbachia* and *Rickettsia*. These yielded no hits in our reference assembly, which is from a member of an all-female brood, and which contained a high-coverage *Spiroplasma* genome. Thus all evidence points toward *Spiroplasma* being the male killer. We note that the ixodetis group of *Spiroplasma* contains known male killers from ladybirds, aphids and a moth.

We have added the blast results to the Supplementary Methods.

Line 227: How is this region “neutral”? Please change the language unless there is compelling support for this.

We have now used the term "colinear" to refer to this portion of the chromosome outside of the BC supergene.

*Also, do you have data indicating spiroplasma was cured? Tetracycline is often required over multiple generations to ensure low titer infections do not persist. This will also eliminate all of the gut/other microbes? Were any steps taken to reseed the microbiome (eg allowing females time on food where other individuals had previously eaten)? It seems unlikely that anything else is influencing sex ratios, but the authors should provide more detail to convince the reader.

We bred the cured line for 6 generations, with the final generations continuing to show normal sex ratios. This is noted in the Methods section. To be more specific, in total across 11 broods and 219 individuals, the sex ratio was 56% female. The cured female we sequenced (a grand-daughter of the treated female) was completely free of *Spiroplasma* reads. This is shown in figure S11 (individual marked with an asterisk. Therefore there is no reason to believe the infection was not eliminated.

We agree that a re-infection experiment will be ideal to prove without doubt that *Spiroplasma* is causal of male killing, but unfortunately that will require further work on site in Africa and has not been achievable yet.

Line 263: What accounts for the 20% reduction? This surprised me and seems much higher than theoretically expected, no?
Nucleotide diversity has two terms: (1) average with-individual sequence divergence (i.e. proportion of heterozygous sites); and (2) average between-individual sequence divergence (averaged across the four possible pairs of sequence comparisons in each pair of individuals). The two terms are weighted according to the total number of within-individual and between-individual comparisons, which means that the second term contributes much more to the total than the first. If each female carries a nearly-identical copy of the neo-W, the second term will be reduced by ~25%, because one in each of the four sequence pairs between a pair of individuals will have ~0 divergence. The first term will be unaffected however, because nobody carries two copies of the neo-W. Hence, a reduction of ~20% is approximately what we would expect.

*Line 285: Based only on reads? Do you have qPCR data? Reads alone are not sufficient in our experience.
Our initial identification of infected individuals was based on depth of reads mapped to the *Spiroplasma* genome. We then performed PCR-based assays on over 100 individuals, including 23 of the genome-sequenced individuals (12 infected and 11 uninfected, Table S12) and these 23 agreed exactly with infection status inferred from read depth. We have added these numbers to the Methods section. Figure S11 shows that infected individuals have clearly non-zero depth on the *Spiroplasma* genome, with most showing over 50x read depth. Jiggins et al. (2000) also showed that PCR reliably detected infection in all broods showing male killing. Although we didn't use a quantitative PCR, the above evidence gives us no reason to suspect that standard PCR is inadequate to detect *Spiroplasma* infection.

*Line 308: B - So the vast majority of nodes have VERY low support? Please report all of the node support and be clear when you can say very little about support for congruence. (I agree with your interpretation, but you should probably be a little more cautious if node support is not great.)

The reviewer is correct that the trees for the neo-W and *Spiroplasma* genome have low bootstrap support. But we disagree with the suggestion that this weakens our result. We have clarified the reasoning in the results section:

"Like the neo-W, the Spiroplasma genomes carry limited variation among individuals (π = 0.0005), consistent with a single and recent outbreak of the endosymbiont. Although the lack of variation makes it challenging to infer genealogies, our inferred maximum likelihood genealogies for the neo-W and Spiroplasma are strikingly congruent (Fig 4B). The low bootstrap support for multiple nodes is unsurprising given that these sequences descend from a recent common ancestor, such that most nodes will be defined by only a few informative sites. This does not weaken the support for congruence however, as the probability of two incorrectly inferred topologies matching by chance is infinitesimally small. In a permutation test for congruence between the two distance matrices [45], the observed level of congruence exceeds all 100,000 random permutations. There is therefore strong support for co-inheritance of the neo-W and Spiroplasma [46]."

We would also like to point out that the permutation test for congruence compares the two distance matrices (as opposed to comparing the two inferred trees), which means that ambiguous relationships that are supported by very few mutations will receive less weight than those that are strongly supported. Nevertheless, all 100,000 permutations of the sample labels failed to produce a stronger correspondence between the two distance matrices than that observed.

Line 348: Add a bit more here because it isn't clear to me why this is support for pre-existing deleterious mutations.
We have expanded this section and also added a statistical test following a suggestion from Reviewer #3. The reasoning should be clearer now:

"Due to purifying selection, non-synonymous polymorphisms are typically rare, and where they do occur the mutant allele typically occurs at low frequency in the population [55]. When considering all polymorphisms in the neo-W lineage, Pn/Ps for chr15 (excluding the BC supergene, to avoid bias) is very slightly (~5%) higher than for other autosomes (Fig S13). Of 1000 bootstrap replicates, 916 reproduced this bias, corresponding to a p-value of 0.084. However, when we partition polymorphisms by allele frequency, we see that chr15 carries a large excess of non-synonymous polymorphisms in the highest frequency class (i.e. minor allele at 50%), with a Pn/Ps ratio >3 times larger than on other autosomes (S13 Fig). This holds across all 1000 bootstrap replicates (i.e. p<0.001). A change in the frequency distribution of non-synonymous variants, without a significant change in their abundance, is best explained by hitchhiking of pre-existing mildly-deleterious alleles that were initially rare in the population but were inadvertently carried to high frequency along with the neo-W haplotype, and are therefore now found in all females in this lineage. In fact, Pn/Ps for high-frequency polymorphisms on chr15 is higher than that for singletons on autosomes (p=0.044), suggesting that accumulation of additional mildly-deleterious alleles on the neo-W might have occurred early during its spread through the population."

*Line 354: What is the overall distribution of spiroplasma in this species/subspecies, and can you say more about why it seems so restricted here? What is expected in terms of spiroplasma spread? Given the time of the association is it surprising that spiroplasma is so geographically restricted here? Other endosymbionts like Wolbachia rapidly spread over a few decades. Is that not expected for spiroplasma? Hurst, Turelli, or others must have relevant theory on this. It seems like there might be something interesting to say given seasonal fluctuations in immigrant males, which are essential given male killing.

We agree that these are very interesting questions. We did speculate briefly on these in the first version, but have now expanded this section as follows:

"The future of the neo-W and Spiroplasma outbreak is uncertain. A lack of males could lead to local extinctions [27], but extinction of the entire infected lineage is unlikely given the high dispersal ability and seasonal influxes of males in the contact zone. Indeed, it is notable that

Spiroplasma infection has only been recorded within the contact zone population (with the exception of a single South African female reported here, S12 Table), especially given theory showing that male killers should spread very rapidly across the geographical range of a panmictic population if they provide even a very weak selective advantage [49]. Future work will investigate whether its spread might be curtailed by environmental factors, for example if oviposition behaviour or host plant availability only leads to sibling competition (and consequent benefits for all-female broods) under certain conditions [45]. An alternative and non-mutually-exclusive hypothesis is that dispersal rates of infected females are strongly reduced."

Line 367: How far do males disperse? How does it vary seasonally (specifically).
Another great question, but sadly one we have no data on currently, apart from that showing seasonal changes in colour morphs (Figure S14).

*Line 459: Is there some justification for the chosen model/approach here? With no partitioning the model assumes everything evolves at the same rate across codon positions. Why not partition the data or assess how assuming rate variation among sites using GTR + G affects the results?

Given that the topologies already matched nearly perfectly when inferred without rate heterogeneity, we did not see a need to use a more complex model. For interest, we have now re-ran all three analyses using a GTR + G model and the topologies were the same. In general, population-level inference tends to be much less dependent on substitution models than deeper phylogenetics where sequences begin to saturate. Indeed most population-level coalescent approaches just use counts of differences.

*Figure 3: The colors in B are difficult at times; specifically, the "x"'s are too light, and distinguishing dorippus and alcippus colors will be difficult for some.

We have now changed the colours throughout the paper to make the different subspecies more easily distinguishable. We also made the Xs indicating associated SNPs bolder.

Reviewer #3:

This manuscript recounts a story of a selfish male-killing bacteria driving the evolution of a neo sex chromosome which also carries a tri-allelic colour polymorphism locus. This work goes some way towards working out the genetics and population genetics of this system, using sequence data and a bit of genetics. Truly fascinating stuff.

The manuscript could use greater clarity and a bit of fleshing out on a few points, however. In particular, it's a very complex system, and I frequently found myself referring back to figure 1, but wishing for more a more comprehensive version of this figure, including information about sample sizes and Spiroplasma infection. A brief overview of the analyses at the beginning of the results would also help.

In general, I found a few comments, e.g., 'To our knowledge, ours is the first example of a butterfly supergene in which the data strongly support the existence of two distinct genes that independently affect colour pattern maintained in LD by suppressed recombination.' strangely defensive. The system is far more interesting that this faint praise would suggest. (Though, as this is purely a stylistic point, I won't complain if they keep this sentence.)

Reviewer #1 also commented on the statement. We have modified it to make the point we were trying to make:

"Our study is one of only a few cases in which it can be shown that alleles at distinct loci that each influence a component of a complex trait are maintained in LD by suppressed recombination [43,44]."

Further, several meiotic drive systems in Drosophila show similar evolutionary patterns (LD between distant loci, most notably in D. pseudoobscura, chromosome-wide hitchhiking), and should be cited where appropriate (see, e.g, Laurracuente et al. 10.1534/genetics.112.141390, Cazemajor GENETICS October 1, 1997 vol. 147 no. 2 635-642, Wu and Beckenbach GENETICS September 1, 1983 vol. 105 no. 1 71-86, Dyer et al. https://doi.org/10.1073/pnas.0605578104).
There is also a similar kind of story in Heliconius currently on bioarxiv doi: https://doi.org/10.1101/736504.
Thank you for these suggestions. We have referenced Palopoli and Wu (1996, Genetics), because it deals explicitly with the extent of hitchhiking around a meiotic drive locus.

Lines 129-137: What are the statistics for the associations mentioned here?
We have clarified in the Methods that this is the Wald, test implemented in PLINK. This is also mentioned in the figure legend (now supplementary figures S2, S5).

Line 188-- 'distinct functional loci' is vague here
This section has been clarified as follows:

"Our study is one of only a few cases in which it can be shown that alleles at distinct loci that each influence a component of a complex trait are maintained in LD by suppressed recombination [43,44]."

Line 220-- what is the evidence for the complete suppression of recombination in females of this species? (I know its thought to be generally true for Lepidoptera, but thought there were exceptions.)

While, in principle, rare female recombination could occur in the wild, we are not aware of any reported cases in the lepidoptera. In our case, the fact that the neo-W haplotype is shared across its entire length by all the females that carry it (indicated by the extremely low genetic diversity), indicates that it has not recombined.

Line 270-- I found the argument that the selection is due to Spiroplasma vs. colour morphs unconvincing: under some models, recessive alleles can spread. In this case, where the frequency of the recessive allele is elevated by linkage to the W, this seems especially true.
We thank the reviewer for highlighting this gap in our reasoning. We have clarified the reasoning as follows:

"The neo-W haplotype carries the recessive BCchrysippus allele at the BC supergene (S4 Fig). However, previous work [22] shows that at the the focal sampling site in the contact zone, most males are immigrants homozygous for the dominant BCdorippus allele, and the vast majority of females (84%) are heterozygous BCdorippus/BCchrysippus , as expected if most inherit BCdorippus from their father and BCchrysippus (on the neo-W) from their mother. The dominant dorippus phenotype is therefore by far the most abundant in this population. Since aposematic colouration should be under positive frequency dependent selection, it is highly unlikely that the

spread of the neo-W can be explained by selection on colour pattern, highlighting the question of what else might have driven its spread"

Figure 3-- having the names repeated over the heterozygotes, with the dominant allele bolded, would help make this figure clearer (particularly when printed in black & white).
Good idea. We have done this. We have also slightly modified the colour scheme so that the alleles are distinguishable in greyscale.

Line 346-- can this prediction be tested quantitatively-- is the Pn/Ps ratio for singletons statistically similar to that seen for the high-frequency mutations? It's a bit hard to tell from figure S11, as the colour scale has no numbers, but it seems like it might be a bit higher, suggesting that there has been some accumulation of mutations.
This is another good suggestion. We have now implemented bootstrapping to allow statistical comparisons. The results are described as follows:

"*When considering all polymorphisms in the neo-W lineage, Pn/Ps for chr15 (excluding the BC supergene, to avoid bias) is very slightly (~5%) higher than for other autosomes (Fig S13). Of 1000 bootstrap replicates, 916 reproduced this bias, corresponding to a p-value of 0.084. However, when we partition polymorphisms by allele frequency, we see that chr15 carries a large excess of non-synonymous polymorphisms in the highest frequency class (i.e. minor allele at 50%), with a Pn/Ps ratio >3 times larger than on other autosomes (S13 Fig). This holds across all 1000 bootstrap replicates (i.e. p<0.001). A change in the frequency distribution of non-synonymous variants, without a significant change in their abundance, is best explained by hitchhiking of pre-existing mildly-deleterious alleles that were initially rare in the population but were inadvertently carried to high frequency along with the neo-W haplotype, and are therefore now found in all females in this lineage. In fact, Pn/Ps for high-frequency polymorphisms on chr15 is higher than that for singletons on autosomes (p=0.044), suggesting that accumulation of additional mildly-deleterious alleles on the neo-W might have occurred early during its spread through the population.*"
