# Supplementary material for: Whole-chromosome hitchhiking driven by a male-killing endosymbiont
Source: PLoS Biol. 2020 Feb 27;18(2):e3000610. doi: 10.1371/journal.pbio.3000610 (PMC7046192; doi:10.1371/journal.pbio.3000610)
Supplement: Attachment — Submitted filename: responses_to_reviewers_2.pdf [file pbio.3000610.s029.pdf]

Reviewer #3:

-Forgive me if I'm being dense, but I'm struggling to understand the meiotic drive argument. As I understand it, the broods of neo-W carrying females are very female biased due to the strong association between the neo-W and the male-killing Spiroplasma (perfect, in the case of the sample in this study). I think the other reviewer perhaps has some Fisherian sex ratio argument in mind, but are they assuming that the neo-W also occurs in non-Spiroplasma infected females (thus reducing the 'base rate' of males)? In any case, I think it would help to flesh out how this system would work for readers (like me) who don't find it intuitive. I do agree that with the larger point of reviewer 1, however, that the added nuance in the discussion has improved the paper, as has the discussion of the sex ratio of cured lines.

-What is the association between the neo-W and Spiroplasma in the wild? (Or, perhaps there's more data for male-killing and the colour polymorphism.) Ten wild-caught females with the right colour pattern and no male killing are document in reference 48-- do we know what proportion this is of the population? Presumably Spiroplasma isn't inherited perfectly, so there should be some neo-W females with no male-killing. If the central hypothesis of the paper is right, however, these should be rare.

To make the argument clearer, we have adjusted two paragraphs in Resulta and Dicussion. First we have clarified the reasoning behind our favoured explanation for the spread of the neo-W:

"We hypothesised that the neo-W has spread as a result of co-inheritance with the male-killing Spiroplasma, which is itself spreading through the population as a selfish element. Experiments have suggested that all-female broods have enhanced survival relative to females from broods that include males, possibly due to reduced competition for resources [45] although other factors such as improved immunity [46] have not been tested. A similar boost to the relative fitness of infected females is thought to have driven the rapid spread of a male-killing Wolbachia in the butterfly Hypolimnas misippus, which has occurred over a similar timescale to that reported here [47]. For Spiroplasma to drive the spread of the neo-W, it would also need to be strictly vertically inherited down the female line, such that it is always co-inherited with the neo-W."

Later in the discussion, we then clarify both the logic of the meiotic drive hypothesis proposed by the other reviewer, and also add more detail on the association between *Spiroplasma* and neo-W, which further shows that this hypothesis is unlikely.

"We cannot entirely rule out the possibility that the neo-W is contributing to this spread, or even driving it entirely, through direct selection or meiotic drive. In theory this is testable by examining broods that carry the neo-W but lack Spiroplasma, as these should comprise more females than males, despite the absence of the male killer. We raised 11 such broods in our cured line, and Smith [50] reported 10 natural broods that showed sex-linked colour pattern and no male killing. Across these 21 broods, totalling 528 adult offspring, 51% were female. This is far from significantly different from the null expectation of 50% (binomial test p=0.7). However, we note that in order to detect meiotic drive causing a 1% female bias with good power would require a far larger sample size of >15,000. Importantly, the few natural broods that have been found to show sex-linked colour pattern without male killing have only been reported from regions in which Spiroplasma infection is present, implying that these broods result from occasional failed-transmission of the endosymbiont [23]. Despite this potential for the neo-W to become decoupled from the male killer, it has not spread beyond these regions, further supporting the hypothesis that hitchhiking with the male killer underlies its rapid spread."

Line 271-- three orders of magnitude?

We have changed this to "more than two orders of magnitude" as 0.00007 is not quite 1000 times lower than 0.0228.

Line 338-- 'this' is ambiguous here; can this sentence be rephrased?

The sentence has been removed in making the changes above.

Line 380-381-- 'higher than for singletons on autosomes' may require a little more explanation. Something like 'higher than expected number of mutations captured on a random copy of an autosome', but more gracefully phrased, would be good.

This section has been rephrased as follows:

"*In fact, P_n/P_s for high-frequency polymorphisms on chr15 is somewhat higher than would be expected through hitchhiking alone based on comparison with singleton mutations on other autosomes (p=0.044). This suggests that accumulation of additional mildly-deleterious alleles on the neo-W might have occurred early during its spread through the population.*"

There are random extra spaces throughout.

These occurred in the in-text references during conversion of the pdf after uploading the source file. All reference formatting marks have now been removed.
